# Multi-Scale Modeling Predicts a Balance of Tumor Necrosis Factor-α and Interleukin-10 Controls the Granuloma Environment during *Mycobacterium tuberculosis* Infection

**DOI:** 10.1371/journal.pone.0068680

**Published:** 2013-07-15

**Authors:** Nicholas A. Cilfone, Cory R. Perry, Denise E. Kirschner, Jennifer J. Linderman

**Affiliations:** 1 Department of Chemical Engineering, University of Michigan, Ann Arbor, Michigan, United States of America; 2 Department of Microbiology and Immunology, University of Michigan Medical School, Ann Arbor, Michigan, United States of America; Tulane University, United States of America

## Abstract

Interleukin-10 (IL-10) and tumor necrosis factor-α (TNF-α) are key anti- and pro-inflammatory mediators elicited during the host immune response to *Mycobacterium tuberculosis* (*Mtb*). Understanding the opposing effects of these mediators is difficult due to the complexity of processes acting across different spatial (molecular, cellular, and tissue) and temporal (seconds to years) scales. We take an *in silico* approach and use multi-scale agent based modeling of the immune response to *Mtb*, including molecular scale details for both TNF-α and IL-10. Our model predicts that IL-10 is necessary to modulate macrophage activation levels and to prevent host-induced tissue damage in a granuloma, an aggregate of cells that forms in response to *Mtb*. We show that TNF-α and IL-10 parameters related to synthesis, signaling, and spatial distribution processes control concentrations of TNF-α and IL-10 in a granuloma and determine infection outcome in the long-term. We devise an overall measure of granuloma function based on three metrics – total bacterial load, macrophage activation levels, and apoptosis of resting macrophages – and use this metric to demonstrate a balance of TNF-α and IL-10 concentrations is essential to *Mtb* infection control, within a single granuloma, with minimal host-induced tissue damage. Our findings suggest that a balance of TNF-α and IL-10 defines a granuloma environment that may be beneficial for both host and pathogen, but perturbing the balance could be used as a novel therapeutic strategy to modulate infection outcomes.

## Introduction

Tuberculosis (TB) is an infectious disease caused by the pathogen *Mycobacterium tuberculosis (Mtb)*. Approximately one-third of the world’s population is infected with *Mtb*, with 2–3 million deaths and an estimated 10 million new clinical cases each year [1], [2]. Upon infection with *Mtb*, 5–10% of individuals develop active pulmonary TB, while about 90% develop a state of chronic infection, known as latent pulmonary TB, showing no clinical signs of disease [3]–[5].

Granulomas are structures which form in the lungs as a result of the immune response to inhaled *Mtb*. Granulomas serve as the central site of host-pathogen interaction during *Mtb* infection, with a host typically developing several granulomas based on the number of inhaled bacteria [4], [6]. During latent pulmonary TB, granulomas are able to control *Mtb* but not completely eradicate the bacteria, while during active pulmonary TB *Mtb* growth is unrestrained in a portion of granulomas. The host factors that control the outcome of infection, in particular the formation and function of a granuloma, are not well understood and thus are difficult to use as therapeutic targets.

**Figure 1:**
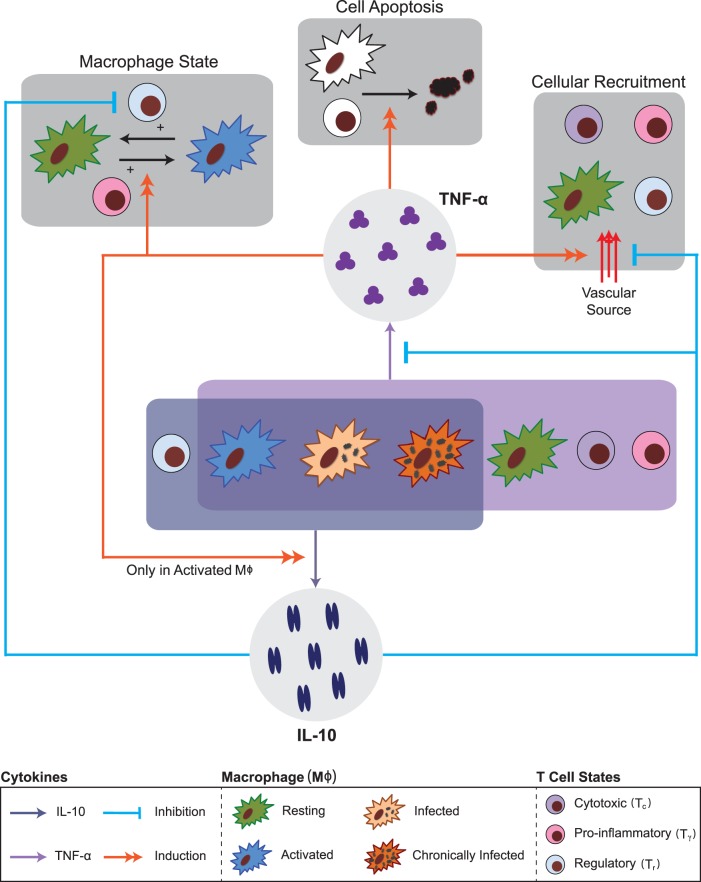
Schematic diagram of TNF-α and IL-10 mechanisms included in GranSim. Regulatory T cells, activated macrophages, infected macrophages, and chronically infected macrophages are able to produce IL-10. IL-10 inhibits the production of TNF-α in all cell types. IL-10 indirectly prevents the recruitment of immune cells to the site of infection by inhibiting chemokine production. IL-10 limits the secondary regulatory mechanism (cell-cell contact, TGF-β, and other regulatory mechanisms) down regulation of activated macrophages by regulatory T cells. Activated macrophages, infected macrophages, chronically infected macrophages, resting macrophages (STAT1 or NFκB activated), cytotoxic T cells, and pro-inflammatory T cells are able to produce TNF-α. TNF-α directly induces recruitment of immune cells to the site of infection (lung). TNF-α induces production of IL-10 in activated macrophages, which represents the pro/anti inflammatory plasticity of activated macrophages. TNF-α, along with interferon-γ derived from pro-inflammatory T cells, induces activation of resting macrophages or it can induce the caspase-mediated apoptosis pathway found in all cell types.

Granulomas have a distinct cellular and spatial organization that creates a unique immune microenvironment in attempt to control infection. Bacteria and infected macrophages are found in the center of the structure and are surrounded by a region of mainly resting and activated macrophages (immune cells that phagocytose foreign material) followed by an outer cuff comprised predominantly of T cells (white blood cells that participate in cell-mediated immunity) [7]–[12]. Formation of a granuloma relies on coordinated immunological processes that include recruitment of immune cells to sites of infection, activation of macrophages, and production of particular molecular mediators known as cytokines [4], [13]–[19]. Cytokines direct immune responses by influencing the fate and behavior of many immune cells. A pro-inflammatory cytokine, tumor necrosis factor-α (TNF-α), and an anti-inflammatory cytokine, interleukin-10 (IL-10), are hypothesized to be central to granuloma formation and function, but understanding the importance of each cytokine is complicated by the myriad of cellular and signaling processes acting across multiple spatial (tissue, cellular, molecular) and temporal (seconds to years) scales (Figure 1) [20]–[25].

IL-10 is a pleiotropic anti-inflammatory cytokine that is produced by immune cells (including both adaptive and innate immune cells) and regulates a variety of immune processes in response to pathogens [20], [22], [23], [25]–[32]. During infection with *Mtb*, IL-10 is primarily produced by infected and non-infected macrophages, with smaller quantities arising from regulatory T cells [20], [23], [25], [33]. Production of IL-10 from other T cell sources, including subsets of CD4+ and CD8+ T cells, is still fairly uncharacterized [20], [34]. IL-10 plays at least three major roles during *Mtb* infection (Figure 1): (1) IL-10 inhibits the production of TNF-α through modulation of STAT3 transcription factors during TNF-α mRNA transcription [35]–[44], (2) IL-10 inhibits the production of chemokines by immune cells, resulting in indirect regulation of cellular recruitment to the site of infection [45]–[48], and (3) IL-10 works in concert with other regulatory mechanisms, such as CTLA-4 and transforming growth factor-β, in order to suppress cellular function, e.g. down regulation of activated macrophages [22], [23], [28], [49]–[51].

Patients with pulmonary TB show elevated levels of IL-10 in lungs, serum, sputum, and bronchoalveolar lavage (BAL) fluid, suggesting a role for IL-10 in preventing control of *Mtb* infection. Genetic studies in humans suggest a correlation between *IL-10* gene polymorphisms and an increase in *Mtb* susceptibility [20]. In *IL-10^−/−^* mice there are reports of enhanced, normal, or poorer control of *Mtb* infection [52]–[62]. Differing genetic backgrounds of the *IL-10^−/−^* mice and differences between mouse models and human infection make these data difficult to interpret. Computational models of *Mtb* infection predict a role for IL-10 in achieving latency with limited tissue damage and in helping balance the major macrophage phenotypes present in granulomas [63], [64]. Finally, in studies of other granulomatous diseases, such as *Leishmania major*, *IL-10^−/−^* mice display severe host damage while IL-10 overexpressing cells show increased recovery from toxic-shock like conditions [26].

TNF-α is a pro-inflammatory cytokine produced by infected and non-infected macrophages, CD4+ T cells, and CD8+ T cells in response to *Mtb* infection [17], [65]. TNF-α mediates multiple immune and bactericidal responses during *Mtb* infection (Figure 1): (1) TNF-α, in conjunction with interferon-γ from CD4+ T cells, activates resting macrophages through the NFκB signaling axis [15], [66]–[70], (2) TNF-α promotes cellular recruitment, both directly and indirectly, by inducing expression of chemokines in macrophages and directly influencing the recruitment of cells from vascular sources [71], (3) TNF-α controls caspase-mediated apoptosis of cells [70], [72], (4) TNF-α can alter the activated macrophage phenotype, thus causing activated macrophages to produce IL-10 [73]–[75]. Studies in both animal and computational models have shown that TNF-α and its controlling processes are critical to the formation and function of granulomas. Removal of TNF-α during *Mtb* infection leads to a range of outcomes, such as unstructured granulomas, and large increases in total bacterial burden [13], [14], [17], [76]–[80]. Furthermore, patients receiving TNF-α antagonists to treat inflammatory diseases such as rheumatoid arthritis show increased incidence of TB reactivation [81], [82].

Taken together, these studies highlight the role of TNF-α as an important initiator of inflammatory and bactericidal processes and IL-10 as an inhibitor of activation and a potential contributor to chronic infection. Several studies have suggested that a balance of TNF-α and IL-10 may be necessary for controlling infection while at the same time preventing severe host tissue damage during *Mtb* infection [5], [20]–[22], [25]. Yet, the complexity of the immune response to *Mtb* (Figure 1) makes it difficult to address this hypothesis using traditional experimental systems.

Here we address the complex and multi-scale effects of TNF-α and IL-10 during *Mtb* infection using a systems biology approach. Building on our previous work [13], we develop a multi-scale computational model of *Mtb* infection that integrates both TNF-α and IL-10 experimental data, including single-cell level receptor-ligand dynamics. We then ask: What molecular scale processes control the concentrations of TNF-α and IL-10? Do TNF-α and IL-10 processes also affect infection outcomes? Does a balance of TNF-α and IL-10 concentrations exist in those granulomas that are able to contain bacteria? Our computational model allows us to explore the dynamics of pro- and anti-inflammatory cytokines across multiple spatial (molecular, cellular, and tissue level) and temporal scales and determine their effects on control of *Mtb* infection.

## Materials and Methods

### Multi-Scale Hybrid Agent-Based Model Overview

**Figure 2:**
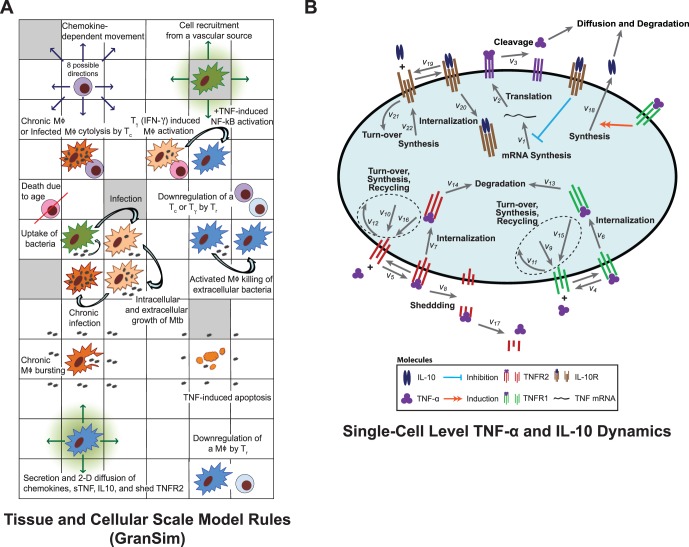
Schematic representation of the hybrid multi-scale ABM of the immune response to *Mtb*. A. An overview of GranSim with a sub-section of model rules shown that represents known immune cell behaviors and interactions (Adapted from [13]). A full list of rules is available in Appendix S1 B. Schematic representation of single cell-level TNF-α and IL-10 binding and trafficking reactions. Model equations are shown in Table S1 in Appendix S2 and Table S2 in Appendix S2.

We constructed a multi-scale computational model of lung granuloma formation and function during *Mtb* infection (Figure S1 in Appendix S1). We describe immune processes over three biological scales: tissue, cellular, and molecular. Tissue and cellular scale events are described with a next-generation two-dimensional agent-based model (ABM) that uses a set of rules and interactions to describe the stochastic behavior of discrete immune cells in the lung (GranSim) [13], [14]. The molecular scale model is composed of three sub-models, each described by systems of differential equations, and is linked to the tissue and cellular scale [83], [84]: (1) Single-cell level TNF-α and IL-10 secretion and receptor-ligand dynamics are described by ordinary differential equations (ODEs), (2) diffusion of TNF-α, IL-10, and chemokines are described by partial differential equations (PDEs), and (3) degradation of TNF-α, IL-10, and chemokines are described by ODEs. Chemokine (consisting of CCL2, CCL5, and CXCL9/10/11) receptor-ligand dynamics, which are not the focus of the current work, are excluded from the model. Figure 2 shows the tissue, cellular, and molecular scale models and how they are interconnected to form our multi-scale hybrid agent-based model of the immune response to *Mtb* infection.

### Tissue and Cellular Scale Model (GranSim)

#### Immune cells and bacilli

We include both macrophages (Mφs) and T cells as distinct agents in our model. These agents have multiple states including: regulatory T cell (T_r_), cytotoxic T cell (T_c_), pro-inflammatory T cell (T_γ_), resting macrophage (resting Mφ), infected macrophage (infected Mφ), chronically infected macrophage (chronic Mφ), and activated macrophage (activated Mφ) (Figure 2A) [14]. Other cell types, e.g. neutrophils, B cells and foamy macrophages, may play a role during *Mtb* infection but are not included in the model due to insufficient evidence as to their function during infection [4]. The model can be easily adapted to include these cell types once more data become available. *Mtb* reside in Mφs but can also exist in the extracellular space of the lung and in areas of hypoxia/caseation [4]. We track three bacterial populations as continuous variables in each Mφ agent or in the extracellular space: intracellular bacteria (B_int_), extracellular bacteria (B_ext_), and bacteria in hypoxic/caseated areas (B_cas_).

#### ABM rules and interactions

Figure 2A shows a representative selection of rules and interactions used to describe the tissue and cellular scale dynamics in the model. The simulation environment consists of 20×20 µm micro-compartments in which agents and bacteria can reside. A full description of rules and interactions is available in Appendix S1. Briefly, rules and interactions describe chemotaxis, intracellular and extracellular growth of *Mtb*, phagocytosis of bacteria by Mφs, T cell killing, interferon-γ induced STAT1 activation of resting Mφs by T_γ_, deactivation of immune cells by T_r_, secretion of chemokines, and development of caseated areas. We updated many agent rules and interactions from our prior generation ABM to reflect additional experimental data including: infected Mφs that transition to activated Mφs kill B_int_ at the same rate that activated Mφs kill B_ext_ [85], [86], resting Mφs that are partially activated (NFκB or STAT1 positive) kill extracellular bacteria at an increased rate [4], [85], [86], partially activated resting Mφs express STAT1 or NFκB activation for a limited timeframe to better describe the need for re-stimulation [69], T cell killing through Fas/FasL or cytotoxic mechanisms occurs in the Moore neighborhood, hypoxic bacterial growth of B_ext_ is scaled based on the number of caseated compartments in the Moore neighborhood [87], and B_cas_ die with a specified death rate and probability [87].

#### Cell recruitment

Recruitment of immune cells to the site of infection has been suggested to be highly correlated with control of *Mtb* [88], [89]. We updated our previous cell recruitment algorithm by adding chemokine- and cytokine-dependent recruitment rates of immune cells to the infection site (Figure S2 in Appendix S1). This is consistent with observed dose-dependent migration rates of immune cells in response to chemokines [88], [90]. We recruit resting Mφs and T cells (T_γ_, T_c_, and T_r_) from vascular sources randomly distributed across the simulation environment. The recruitment rate at each vascular source is dependent upon the concentrations of CCL2, CCL5, CXCL9, and TNF-α in the specified micro-compartment. Recruitment rates for each molecule are modeled using a concentration threshold and Michaelis-Menton kinetics. The overall recruitment rate for each agent is the summation of the contribution by CCL2, CCL5, CXCL9, and TNF-α and is scaled by a maximum probability of recruitment, which turns the continuous recruitment rate into a stochastic event. The probability functions for recruitment of resting Mφs, T_γ_, T_c_, and T_r_ are given in Appendix S1 and values of recruitment parameters are given in Table S3 in Appendix S3.

### Molecular Scale Model

#### IL-10 and IL-10 receptor-ligand dynamics

We developed a model of IL-10 receptor-ligand dynamics based on experimental data (Figure 2B). We assume that IL-10 is synthesized by infected Mφs, chronic Mφs, activated Mφs, and T_r_ and released directly into the extracellular environment [25], [26], [34], [50], [91]–[95]. IL-10 exists in the extracellular space as a non-covalently bonded dimer where it can bind to cell-surface IL-10R1 and IL-10R2 [96], [97]. Signaling occurs through association of bound IL-10R1 with the IL-10R2 subunit. IL-10R1 is the high affinity receptor compared to IL-10R2, which mainly exists as a signaling subunit to bound IL-10R1 [26]. For simplicity, we include only a general IL-10R that represents both IL-10R1 and IL-10R2 [98], [99]. IL-10R is synthesized by cells and is removed from the membrane by turnover [100]. Bound IL-10R can be internalized followed by degradation or recycling to the surface [101]. These processes are modeled by mass-action kinetics as shown in Table S1 in Appendix S2 and Table S2 in Appendix S2; definitions and values of rate constants are defined in Table S4 in Appendix S3.

#### TNF-α and TNF-α receptor-ligand dynamics

TNF-α and TNF-α receptor-ligand dynamics events are illustrated in Figure 2B. The equations describing these events were modified from earlier work in order to accommodate IL-10 receptor-ligand dynamics as discussed above [13], [80], [102]. TNF-α mRNA is transcribed and subsequently translated into its membrane bound form, mTNF, by NFκB-activated resting Mφs, infected Mφs, chronic Mφs, activated Mφs, T_γ_, and T_c_. mTNF is cleaved from the membrane into its soluble form (sTNF), by the metalloproteinase TACE. sTNF can reversibly bind to the two receptors, TNFR1 and TNFR2. TNFR1 and TNFR2 are synthesized by the cell and are removed from the membrane by turnover. Bound TNFR1 and TNFR2 can be internalized and then degraded or recycled to the surface in their unbound state. sTNF bound to TNFR2 may be shed to the extracellular space where it can dissociate. These processes are modeled by mass-action kinetics as shown in Table S1 in Appendix S2 and Table S2 in Appendix S2; definitions and values of rate constants are defined in Table S4 in Appendix S3.

#### Linking TNF-α and IL-10 receptor-ligand dynamics

TNF-α and IL-10 receptor-ligand dynamics are linked in two ways (Figure 2B): bound IL-10R inhibits TNF-α mRNA transcription and bound TNFR1 can induce synthesis of IL-10 in activated Mφs [35], [73]. Inhibition of TNF-α mRNA transcription shows rapid switch-like behavior, and we modeled this process with a three-parameter logistic function [35]:
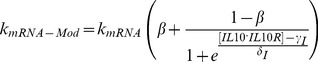
(1)


We captured the ability of bound TNFR1 to induce synthesis of IL-10 in activated Mφs with Michaelis-Menton type kinetics, which roughly approximates the mechanisms influencing the plasticity of activated Mφs to produce IL-10 at lower (classically activated Mφs) or higher (alternatively activated Mφs) rates [73]–[75], [103], [104].
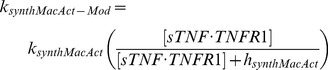
(2)


Parameter values and definitions are found in Table S4 in Appendix S3. Other molecular scale mechanisms of IL-10 inhibition of TNF-α processes, such as inhibition of TACE activity, can be included in future models as more data become available [37], [105], [106].

#### Diffusion of soluble molecules

Diffusion of soluble TNF-α, IL-10, and chemokines is described by the two-dimensional diffusion equation:
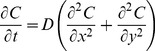
(4)


We track soluble CCL2, CCL5, CXCL9, TNF-α, IL-10, and shed bound TNFR2 as continuous species that can diffuse in the lung environment. We use the alternating-direction explicit (ADE) method for discretizing the environment and solving the diffusion equation [107]. This method is unconditionally stable, allowing us to use a solver time step five times larger than in previous work, simultaneously increasing computational performance and solution accuracy (Appendix S2).

#### Degradation of soluble molecules

Degradation of soluble CCL2, CCL5, CXCL9, TNF-α, IL-10, and shed bound TNFR2 is described by:
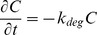
(5)


In order to increase the accuracy of the degradation calculation and to prevent unnecessary computational burden, we calculate degradation using the analytical solution to Eq. 5 (Appendix S2).

### Linking the Molecular Scale Model to the Tissue and Cellular Scale Model

Molecular scale dynamics involving TNF-α are linked to the tissue and cellular scale model (GranSim) (Figure 2) through cell recruitment (discussed above), NFκB activation of Mφ, and caspase induced cell apoptosis. We describe TNF-α induced NFκB activation of each Mφ and TNF-α induced apoptosis of cells as Poisson processes with a probability of occurrence determined by a rate constant, threshold value, and a saturation value (see Eq. 8 and 9 in Appendix S2 for equations and Table S5 in Appendix S3 for parameter definitions and values). TNF-α induced NFκB activation of Mφ is dependent on the concentration of bound TNFR1 per cell, while TNF-α induced apoptosis is dependent on the concentration of internalized bound TNFR1 per cell [13], [102]. Molecular scale dynamics involving IL-10 are linked to GranSim through chemokine down regulation and compensation of alternative suppressive functions. IL-10 inhibits the production of chemokines by Mφs; we use a simple threshold relationship in which the synthesis of chemokines is reduced by half once the number of bound IL-10R is above the specified threshold [45]–[48]. The probability of alternative suppressive functions of T_r_ occurring is linearly dependent on the ratio described by:
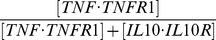
(6)


This simulates the dependence of other regulatory mechanisms on IL-10 that are not the focus of this work [22], [23], [28], [49]–[51].

### Parameter Estimation and Model Validation

We estimate model parameter values using experimental data where available and previous modeling studies [13], [14], [63]. When data are unavailable we use uncertainty and sensitivity analyses to explore the parameter space to match observed qualitative behavior (see below) [108]. Cell-specific TNF-α receptor-ligand dynamics model parameters are estimated from literature as previously described [13], [80], [102]. We updated parameter estimates for rate constants, concentration thresholds, and saturation concentrations of TNF-α induced Poisson processes [69]. Parameters for the updated cell recruitment algorithm were estimated using uncertainty and sensitivity analyses and validated by comparing total T cells numbers against non-human primate estimates [109].

IL-10 receptor-ligand dynamics model parameters are estimated based on experimental data. The synthesis rate constant of IL-10 by infected Mφs is estimated using *in vitro* kinetic IL-10 ELISA data from both human monocyte-derived Mφs and a THP-1 cell line infected with *Mtb* strain H37Rv [92], [95]. The synthesis rate constant of IL-10 by activated Mφ is estimated using *in vitro* kinetic IL-10 ELISA data from M-CSF activated human monocyte-derived Mφs and single-time point IL-10 ELISA data from interferon-γ activated murine Mφ [91], [104]. The half-saturation of IL-10 induction by bound TNFR1 in activated Mφs is estimated using uncertainty and sensitivity analysis such that on average synthesis rates of IL-10 fall into above estimated ranges. Synthesis of IL-10 by T_r_ is estimated using *in vitro* kinetic IL-10 ELISA data from both human and mouse purified T cells [34], [93], [94]. The three parameters describing inhibition of TNF-α mRNA synthesis by bound IL-10R (Eq. 1), are estimated from *in vitro* human monocyte-derived Mφs stimulated with *Mycobacterium avium* (cultured with and without an anti-IL-10 antibody) or stimulated with lipopolysaccharide [36], [37]. Data on the peak, timing, and shape of soluble TNF-α concentration data were used to estimate parameters describing TNF mRNA synthesis (See Figure S4 in Appendix S4). A complete list of IL-10 and TNF-α receptor-ligand dynamics parameters along with all other model parameters are found in Table S3 in Appendix S3, Table S4 in Appendix S3, and Table S5 in Appendix S3.

Using methods described above we determine a baseline set of parameters, referred to as the *baseline containment parameter set*, which results in robust control of *Mtb* infection with structures similar to granulomas observed in humans and non-human primates. We validate and test our baseline containment parameter set using multiple experimentally verified scenarios to ensure it can describe the immune response to *Mtb* infection. For example, we perform virtual deletion simulations that mimic experimental studies of gene knockouts by setting the relevant model parameters (probabilities and rate constants) to zero from the beginning of a simulation. Virtual deletion experiments are carried out for interferon-γ, TNF-α, and T cells for model validation, while IL-10 deletion experiments are predictions of the model. We compare our predicted simulations with corresponding data from human, mouse, and non-human primate studies.

### Model Outputs and Analysis Metrics

We consider multiple model outputs relevant to granuloma formation and function during *Mtb* infection. At the cellular scale, we measure numbers of immune cells, frequency of T cell killing, caseated areas, and rates of cellular recruitment. At the molecular scale, we track average concentrations of TNF-α and IL-10 in the granuloma along with number of TNF-α induced processes. Specifically, we measure the number of resting Mφs that undergo TNF-α induced apoptosis as a metric of healthy tissue damage, the number of activated Mφ as a metric of bactericidal activation levels, and total bacterial load (the sum of B_int_ and B_ext_) as a metric of infection status. Simulation results are organized by infection outcome, which includes bacterial containment (stable total bacterial load of ∼10^3^), clearance (total bacterial load of zero), and unresolved clearance (total bacterial load of zero with non-steady state numbers of activated Mφs), and data is shown for only the dominant outcome. This allows us to use common statistical techniques to analyze the results. Further explanation and non-dominant outcome data is provided in Appendix S4.

We hypothesize that a beneficial outcome for the host corresponds to a low total bacterial load, low tissue damage (induced by apoptosis), and sufficient immune activation. Thus, we combine the three metrics – total bacterial load, healthy tissue damage, and Mφ activation levels – together into a single metric we define as the *host-pathogen index (H.P.I.)*.
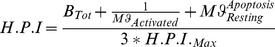
(7)


H.P.I. is an evenly weighted average of the total bacterial load, the number of apoptotic resting Mφs, and the inverse of the number of activated Mφs, normalized between zero and one. The inverse of the number of activated Mφs is used so that low values for all metrics correspond to beneficial behavior.

To simplify the presentation of the influence of molecular scale TNF-α and IL-10 related parameters we divide them into three groups for each molecule: (1) parameters that are part of the synthesis pathway are organized into the *synthesis group*, (2) parameters that control the spatial aspects are organized into the *spatial group*, and (3) parameters that control binding and signaling are organized into the *signaling group*. Table S4 in Appendix S3 and Table S5 in Appendix S3 describe each parameter and group.

### Uncertainty and Sensitivity Analysis

Uncertainty and sensitivity analyses are used to identify model parameters that have a significant effect on model output. Latin hypercube sampling (LHS) is a straightforward and computationally effective method for simultaneously varying multiple parameters and sampling the parameter space [108]. Partial rank correlation coefficients (PRCCs) are used to quantify the effects of varying each parameter on non-linear model outputs, indicating model sensitivity to specific parameters. A PRCC value of -1 is a perfect negative correlation while a PRCC value of +1 represents a perfect positive correlation. PRCC values are differentiated with a student t-test to assess significance. In this work, we use the LHS algorithm to generate 250 unique parameter sets, which are simulated in replication 4 times and the average of the outputs are used to calculate PRCC values. This number of simulations indicates a PRCC value of above 0.24 or below -0.24 is considered to be highly significant with a p value <0.001 [108]. We separate the molecular scale TNF-α and IL-10 model parameters from GranSim model parameters when carrying out LHS in order to better understand how each scale affects the others (intra vs. inter-scale effects).

### Model Implementation

A two-dimensional 100×100 lattice of micro-compartments represents a 2×2mm section of lung tissue. Initial conditions include pre-seeded resting Mφ in the lung environment and placing an infected Mφ at the center of the grid [6]. We use zero concentration boundary conditions for diffusion of soluble molecules on all four of the grid edges. Granulomas are typically separated from one another and tissue concentrations of chemokines and cytokines are typically negligible in normal lung tissue [6]. We use torodial boundary conditions for agent movement on the lattice. The rules and interactions of the ABM are solved on a 10 min time step (28,800 agent time steps for 200 days of simulation). Diffusion and degradation of soluble molecules are updated on a 30 second time step. TNF-α and IL10 receptor-ligand dynamics ODEs are solved for each agent using a Runge-Kutta 4^th^ order numerical solver with a time-step of 6 seconds.

Our model was constructed using the C++ programming language in conjunction with Boost libraries (distributed under the Boost Software License – available at www.boost.org). The Qt framework (open-source, distributed under GPL – available at qt.digia.com) was used to build the graphical user interface (GUI), which allows us to visualize, track, and plot different facets of our simulated granulomas in real-time. The model can be used with or without GUI visualization and is cross-platform (Mac, Linux, Windows). Simulations were performed on the Nyx/Flux computing cluster available at the Center for Advanced Computing at the University of Michigan and post-processing for visualization was carried out on non-cluster resources, which included multi-core desktops and laptops.

## Results

### Simulated Granulomas Display Infection Outcomes Comparable to Experimental Models

**Figure 3:**
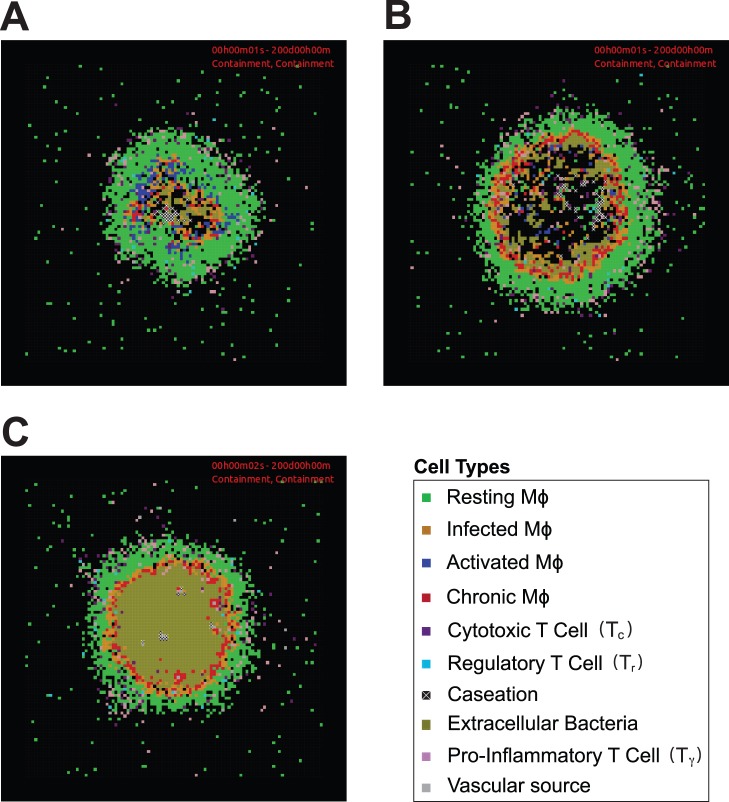
Model validation of simulated granulomas at 200 days post-infection. A. Simulation using baseline containment parameter set. B. Simulation using TNF-α knockout parameter set (baseline containment parameter set but with k_RNA_Mac_ = 0 and k_RNA_Tcell_ = 0). C. Interferon-γ knockout parameter set (baseline containment parameter set P_STAT1_ = 0). Cell types are as follows: resting macrophages (resting Mφ), infected macrophages (infected Mφ), chronically infected macrophage (chronic Mφ), activated macrophage (activated Mφ), pro-inflammatory T cell (T_γ_), cytotoxic T cell (T_c_), regulatory T cell (T_r_), and extracellular bacteria (B_ext_). Agent and bacteria colors are shown in the included legend. These same colors are used for subsequent images. Model parameters are given in Table S3 in Appendix S3, Table S4 in Appendix S3, and Table S5 in Appendix S3. For full length time-lapse simulations please see http://malthus.med.micro.umich.edu/lab/movies/TNF-IL10.

We first test whether our multi-scale computational model can recapitulate known features of the immune response to *Mtb* infection. Using estimation methods described above, we identify a *baseline containment parameter set* (Table S4 in Appendix S3 and Table S5 in Appendix S3) that leads to robust control of *Mtb* infection over 200 days post-infection, including a stable total bacterial load of ∼10^3^ and organized granulomas comparable to histological observations in humans and non-human primates [7]–[10]. Simulated granulomas (Figure 3A) contain a core of infected Mφs, chronic Mφs, B_ext_ and areas of caseation that are surrounded primarily by resting Mφs and activated Mφs, and a peripheral region consisting mainly of T_c_, T_γ_, and T_r_. About 15% of simulations using the baseline containment parameter set lead to granulomas that are able to clear all bacteria. This occurs when infected Mφs and chronic Mφs are killed through TNF-α induced apoptosis or T cell mediated killing at the initial stages of simulated infection. Data suggest that some individuals do not become infected when exposed to *Mtb*, consistent with our simulations that result in clearance before a granuloma begins to form [16]. By varying model parameters in the baseline containment parameter set our model is also able to predict different granuloma outcomes, as observed in non-human primate models, including primarily clearance or uncontrolled growth of bacteria [16].

We further validate our model by performing virtual deletion experiments (see Materials and Methods) for TNF-α (k_RNA_Mac_ = 0 and k_RNA_Tcell_ = 0) and interferon-γ (P_STAT1_ = 0). These lead to an increased total bacterial load including increased B_ext_ in the interior of larger and more irregular shaped granulomas (Fig. 3B–C). TNF-α and interferon-γ are important initiators of inflammatory and bactericidal processes during *Mtb* infection [4]. Virtual deletions of TNF-α or interferon-γ are unable to control disease progression due to a lack of Mφ activation and bactericidal activity, consistent with experimental data [16], [17]. These results also agree with our previous computational studies using models that did not include molecular level dynamics for IL-10 [13], [14].

### IL-10 is Necessary to Control Inflammatory Processes and Tissue Damage during *Mtb* Infection

**Figure 4:**
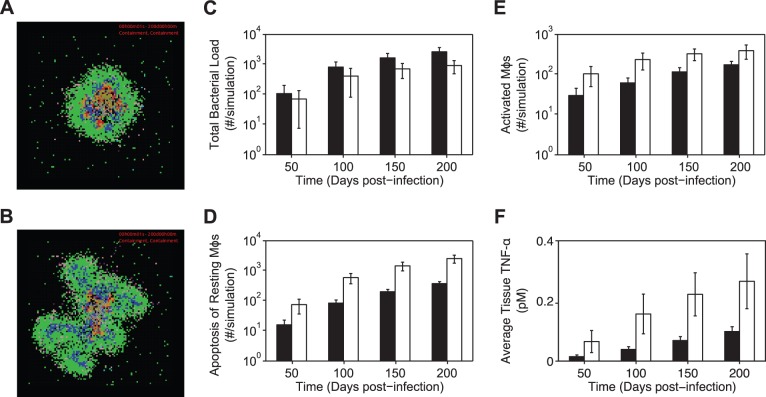
Time course simulation results for baseline and IL-10 knockout scenarios. Simulation using baseline containment parameter set at 200 days post-infection. B. Simulation using the IL-10 knockout parameter set at 200 days post-infection. Agents and bacteria colors are as in Figure 3. C–F. Simulation results at 50, 100, 150 and 200 days post-infection using the baseline containment parameter set (black bars) and the IL-10 knockout parameter set (white bars). The few simulations that lead to clearance of *Mtb* in a granuloma are not shown here (see Table S10 in Appendix S4). C. Total bacterial load. D. Number of activated Mφ. E. Number of apoptotic resting Mφ. F. Average tissue concentration of TNF-α (pM). For full length time-lapse simulations please see http://malthus.med.micro.umich.edu/lab/movies/TNF-IL10.

To explore the role of IL-10 during *Mtb* infection, we probe both the formation and function of granulomas when IL-10 is absent. We perform a virtual deletion experiment by setting IL-10 synthesis parameters to zero in the baseline containment parameter set (k_synthMacAct_ = 0, k_synthMacInf_ = 0, and k_synthTcell_ = 0), thus generating the *IL-10 knockout parameter set*. Figure 4 shows the average total bacterial load, number of activated Mφs, apoptosis of resting Mφs, and average tissue TNF-α concentration for simulations using either the baseline containment parameter set or the IL-10 knockout parameter set.

IL-10 knockout (*IL-10^−/−^*) granulomas have similar total bacterial loads compared to baseline containment granulomas, but with elevated numbers of activated Mφs, increased apoptosis of resting Mφs, increased cellular infiltration rates (not shown), and increased average tissue TNF-α concentrations (Figure 4C–F). *IL-10^−/−^* granulomas contain large clusters of activated Mφs in the periphery of the structure indicating that they fail to maintain proper levels of immune cell activation (Figure 4A–B). *IL-10^−/−^* granulomas show an increased probability of clearing *Mtb*, with ∼40% of simulations clearing bacteria compared to ∼15% of simulations with the baseline containment parameter set. However, the increase in clearance outcomes is also associated with large clusters of activated Mφs and increased apoptosis of resting Mφs that persists beyond clearance of bacteria (see Table S10 in Appendix S4). The excessive influx of inflammatory cells, in both *IL-10^−/−^* containment and clearance outcomes, is directly due to the lack of IL-10, which controls multiple inflammatory processes through multiple mechanisms (Figure 1). Thus, our model suggests that IL-10 is necessary during *Mtb* infection to modulate activation levels and restrict apoptosis of resting Mφs (a measurement of tissue damage), which is consistent with earlier modeling studies [63], [64]. These results also suggest that variable outcomes reported from experimental *IL-10^−/−^* studies may be due to the large amount of variability in infection outcome, small sample sizes, and differences between animal models [52]–[62].

### Granuloma Outcomes are Sensistive to Multiple TNF-α and IL-10 Processes that Control Average Concentrations in a Granuloma

**Figure 5:**
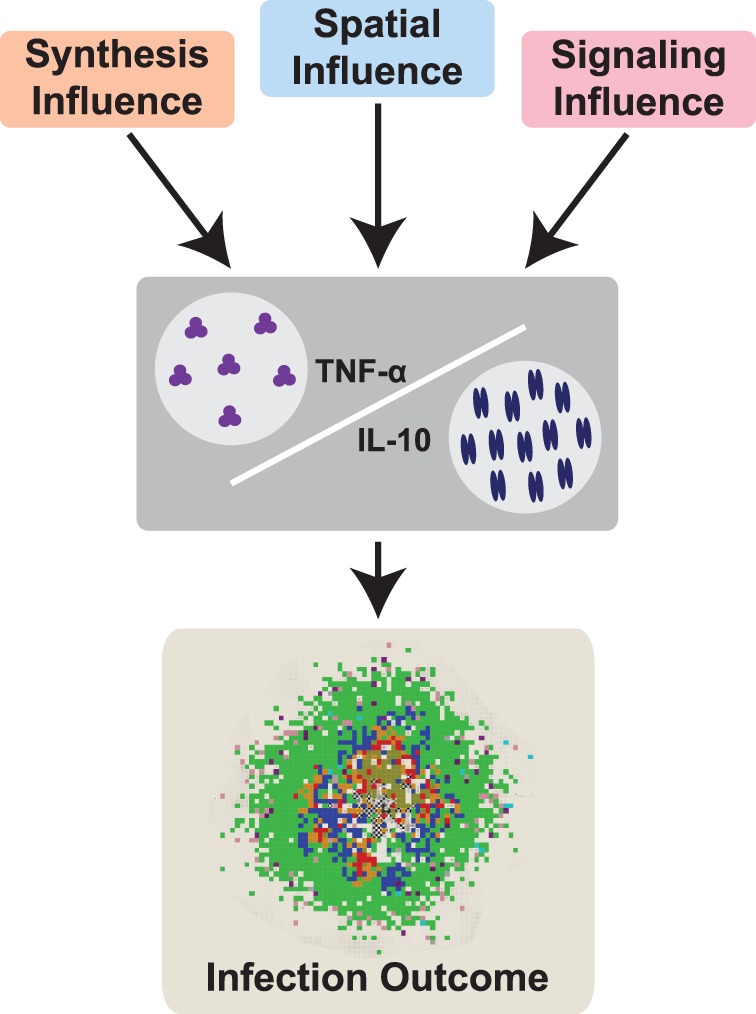
Three main processes influence the concentrations of TNF-α and IL-10 and control infection outcome. Model parameters that are relevant to TNF-α or IL-10 synthesis (synthesis influence), that control the spatial distribution of TNF-α or IL-10 (spatial influence), and that control the binding and signaling of TNF-α or IL-10 (signaling influence). These three parameter groups control the concentrations of TNF-α and IL-10 in the granuloma environment and thus in turn directly control infection outcome. Parameter groups are described in Table S4 in Appendix S3 and Table S5 in Appendix S3.

We now predict which biological processes are controlling concentrations of TNF-α and IL-10 in a granuloma during *Mtb* infection. We analyze the impact of TNF-α and IL-10 molecular scale processes on granuloma outcomes using sensitivity analyses and group important parameters together based on the relevant processes they describe. Table 1 lists model parameters that are significantly correlated with key features of the granuloma: total bacterial load, average tissue concentration of TNF-α, average tissue concentration of IL-10, and granuloma size (see also Table S11 in Appendix S4 and S12 in Appendix S4). These important TNF-α and IL-10 parameters fall into three groups (as defined in Materials and Methods): (1) the *synthesis group*, (2) the *spatial group*, and (3) the *signaling group*. Processes described by the parameters in these three groups shape the concentrations of TNF-α and IL-10 in a granuloma environment and thus in turn control granuloma outcome (Figure 5). We now focus our analyses on the effects of manipulating parameters in each of the three groups on granuloma formation and function.

**Table 1:** Molecular Scale TNF-α and IL-10 Model Parameters Significantly Correlated With Selected Model Outputs At 200 Days Post-Infection.

			Selected Model Outputs†			
Parameter Group	Parameter	Parameter Description	TotalBacterialLoad*	AverageTissueTNF-α	AverageTissueIL-10	GranulomaSize
TNF-α Synthesis	k_SynthMac_	Minimum TNF-α mRNA synthesis rate of macrophages	−	+		
	k_RNA_Mac_	Basal TNF-α mRNA synthesis rate of macrophages	−	+++		
TNF-α Signaling	K_d1_	Equilibrium dissociation constant of sTNF/TNFR1	+++			
	k_on1_	sTNF/TNFR1 association rate constant	−			
	k_int1_	TNFR1 internalization rate constant	+++	−	−	−
	k_t2_	TNFR2 turn-over rate constant	−			
	TNFR1_Mac_	TNFR1 density on the surface of macrophages	−	−		−
	τ_apop_	Internalized sTNF/TNFR1 threshold for TNF-induced apoptosis	++			
	τ_NFkB_	Cell surface sTNF/TNFR1 threshold for TNF-induced NFκB activation		−	−	−
	k_NFkB_	Rate constant for TNF-induced NFκB activation in macrophages	−			
TNF-α Spatial	D_TNF_	Diffusion coefficient of sTNF		+++	++	+++
	k_deg_	sTNF degradation rate constant	++	−	−	
IL-10 Synthesis	k_SynthMacAct_	Full synthesis rate of IL-10 by activated macrophages	+++	−		−
IL-10 Signaling	K_d_	Equilibrium dissociation constant of IL-10/IL-10R	−			
	k_on_	IL-10/IL-10R association rate constant	+++	−	−	
	k_int_	IL-10R internalization rate constant	−	+++	+	
	IL10R_Mac_	IL-10R density on the surface of macrophages	+++	−	−	
IL-10 Spatial	k_deg_	Soluble IL-10 degradation rate constant	−	++	−	

Significant PRCC values are as follows: −/+0.001<p<0.01−/++0.0001<p<0.001−/+++ p<0.0001.

* Bacterial load incorporates PRCC values for IntMtb, ExtMtb, and TotMtb.

† Detailed sensitivity analysis is presented in Table S11 in Appendix S4 and S12 in Appendix S4.

### Synthesis Rates of TNF-α and IL-10 have Opposing Effects on Bacterial Control and Tissue Damage

We first analyze the effects of parameters in the synthesis group on a granuloma environment. Sensitivity analysis (Table 1) indicates that the basal rate of TNF-α mRNA synthesis by Mφs correlates negatively with total bacterial load and positively with average tissue TNF-α concentration, while the IL-10 synthesis rate for activated Mφs correlates positively with total bacterial load and negatively with average tissue TNF-α concentration. Thus, TNF-α and IL-10 synthesis group parameters have a significant impact on their concentrations in a granuloma environment.

**Figure 6:**
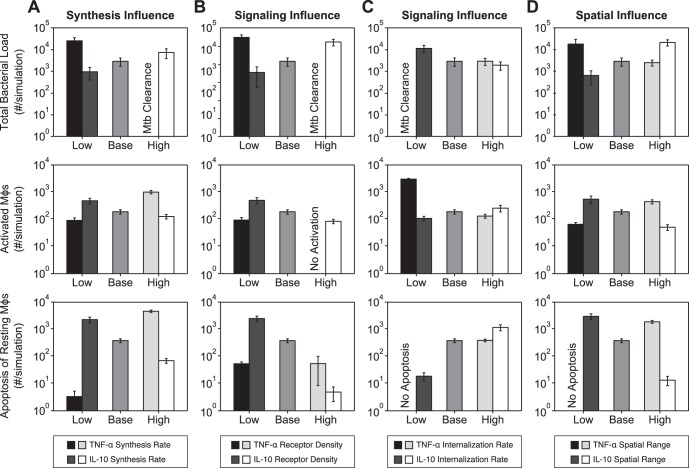
Simulation results showing the effects of varying each influence in a granuloma environment. Simulation results from 30 replications showing the effects of varying each of the three influences: the synthesis influence, signaling influence, and spatial influence (Figure 5; Table S4 and Table S5 in Appendix S3). Results using the baseline containment parameter set, labeled ‘base’, are included for comparison (parameter values in Table S4 in Appendix S3 and Table S5 in Appendix S3). A. Effects of mRNA synthesis rate of TNF-α by Mφ (‘Low’ *k_RNA_Mac_* = 0.5 #/cell*s, ‘High’ *k_RNA_Mac_* = 3.0 #/cell*s) and synthesis rate of IL-10 by activated Mφ (‘Low’ *k_synthMacAct_* = 0.1 #/cell*s, ‘High’ *k_synthMacAct_* = 1.0 #/cell*s). B. Effect of TNFR1 receptor density on Mφ (‘Low’ *TNFR1_mac_* = 500, ‘High’ *TNFR1_mac_* = 5000) and IL-10R receptor density on Mφ (‘Low’ *IL10R_mac_* = 500, ‘High’ *IL10R_mac_* = 5000). C. Effect of bound TNFR1 internalization rate constant (‘Low’ *k_int1_* = 10^−4^ s^−1^, ‘High’ *k_int1_* = 10^−3^ s^−1^) and bound IL-10R internalization rate constant (‘Low’ *k_int_* = 10^−4^ s^−1^, ‘High’ *k_int_* = 10^−3^ s^−1^). D. Effect of spatial range of TNF-α (‘Low’ *D_TNF_* = 1×10^−8^ cm^2^/s *k_deg_* = 2.3×10^−2^ s^−1^, ‘High’ *D_TNF_* = 9×10^−8^ cm^2^/s *k_deg_* = 5×10^−5^ s^−1^) and spatial range of IL-10 (‘Low’ *D_IL10_* = 1×10^−8^ cm^2^/s *k_deg_* = 1.6×10^−2^ s^−1^, ‘High’ *D_IL10_* = 8×10^−8^ cm^2^/s *k_deg_* = 1.8×10^−6^ s^−1^). Low indicates a lower value than baseline while high indicates a higher value than baseline.

We manipulate the synthesis rates of TNF-α mRNA by Mφs and the synthesis of IL-10 by activated Mφs to both high and low values. Figure 6A shows the results of these manipulations on average total bacterial load, number of activated Mφs, and apoptosis of resting Mφs (see also Table S9 in Appendix S4). Low rates of TNF-α mRNA synthesis and high rates of IL-10 synthesis show increased bacterial loads with lower levels of activated Mφs and less apoptosis of resting Mφs. Conversely, high rates of TNF-α mRNA synthesis and low rates of IL-10 synthesis display clearance or reduced bacterial loads with increased levels of activated Mφs, but at the cost of increased apoptosis of resting Mφs. Thus, the rates of synthesis of TNF-α mRNA and IL-10 have opposing effects on controlling bacterial load and on preventing tissue damage in the host. Using moderate synthesis rates for TNF-α and IL-10 (as reflected in our baseline containment parameter set) results in bacterial containment with limited host-mediated tissue damage in a granuloma.

### Signaling Parameters Establish the Best Response to TNF-α and IL-10 Concentrations that Regulates Apoptosis and Activation of Macrophages

Next we explore the effects of parameters in the signaling group on granuloma formation and function to understand how signaling parameters control the response to concentrations of TNF-α and IL-10 in a granuloma environment (Figure 5). Sensitivity analysis (Table 1) reveals that the internalization rate constants of bound TNFR1 and bound IL10R1 and the average densities of TNFR1 and IL-10R1 on Mφ are significantly correlated with total bacterial load and average tissue concentrations of TNF-α and IL-10 (see also Table S6 in Appendix S4 and Table S8 in Appendix S4). Hence, we analyze the effects of changing *two* signaling parameters, for both TNF-α and IL-10, on average total bacterial load, number of activated Mφs, and apoptosis of resting Mφs.

Figure 6B shows simulation results for manipulations of TNFR1 density and IL-10R density on the surface of Mφs (see also Table S6 in Appendix S4). Low TNFR1 densities lead to increased bacterial loads, lower levels of activated Mφs and less apoptosis of resting Mφs by preventing Mφs from responding to the concentration of TNF-α in a granuloma environment. The benefit in reducing TNF-α induced apoptosis is outweighed by the decrease in TNF-α induced NFκB activation. High TNFR1 densities lead to bacterial clearance with limited activated Mφs and low apoptosis of resting Mφs. Increased TNFR1 density promotes the ability of infected Mφs to respond to the concentration of TNF-α, which leads to early activation and apoptosis of infected cells and encourages bacterial clearance. We observe lower total bacterial numbers with higher levels of activated Mφs and increased apoptosis of resting Mφs at low IL-10R densities, resulting from the decreased response of immune cells to IL-10 concentrations. The reduced response decreases the likelihood of initiating the IL-10 inhibition cascade and limits TNF-α production and its induced processes. High IL-10R densities promote inhibitory signaling, leading to an increased bacterial load with less activated Mφs and limited apoptosis of resting Mφs.

Next, we manipulate the internalization rate constants of bound TNFR1 and bound IL-10R (Figure 6C, see also Table S8 in Appendix S4). Low internalization rates of bound TNFR1 cause bacterial clearance with no apoptosis of resting Mφs, but with uncontrolled numbers of activated Mφs since low internalization rates favor NFκB induction over apoptosis as the response to TNF-α concentrations [67], [78]. High internalization rates of bound TNFR1 have limited effects on total bacterial load and apoptosis of resting Mφs with only a slight decrease in number of activated Mφs. Lowering the internalization rate of bound IL10R leads to an increased bacterial load, decreased apoptosis of resting Mφs, and no change in activated Mφs, while increasing the internalization rate only increases apoptosis of resting Mφs with no changes in total bacterial load or activated Mφs. These results suggest that the internalization rate of bound IL-10R is preventing apoptosis of resting Mφs at the cost of bacteria load. Taken together, these results suggest that the parameters in the signaling group are establishing the most beneficial response to the concentrations of TNF-α and IL-10, which is critical to the control of the immune response occurring in a granuloma.

### TNF-α and IL-10 Spatial Parameters Focus Bactericidal Processes in Infected Regions of a Granuloma and Limit Healthy Tissue Damage in Non-infected Regions

Lastly, we explore the effects of parameters in the spatial group on granuloma formation and function (Figure 5). Sensitivity analysis (Table 1) demonstrates that diffusivity of TNF-α and degradation rates of TNF-α and IL-10 correlate with average tissue concentrations of TNF-α and IL-10 and total bacteria load (see also Table S11 in Appendix S4 and Table S12 in Appendix S4). These results highlight that the spatial distributions of TNF-α and IL-10 are important for granuloma function. To probe the role of the spatial distribution of cytokines, we simultaneously manipulate the diffusivity (D) and degradation rate (k_deg_) of TNF-α or IL-10 in order to extend or contract the spatial range (L) of each molecule, as estimated by [110] (see also Figure S3 in Appendix S4):
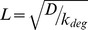
(8)


Figure 6D shows the effects of altering the spatial range of TNF-α and IL-10 on average total bacterial load, number of activated Mφs, and apoptosis of resting Mφs (see also Table S7 in Appendix S4).

Reduced TNF-α spatial ranges lead to an increased total bacterial load with a decrease in activated Mφs and limited apoptosis of resting Mφs. Increased TNF-α spatial ranges cause no change in total bacterial load, small changes in activated Mφs and significant increases in apoptosis of resting Mφs. These results suggest that if the spatial range of TNF-α is large, the response of resting Mφs in the periphery of granulomas shifts from NFκB activation to apoptosis. Similarly, if the spatial range of TNF-α is insufficient, apoptosis of infected Mφ in the core of the granuloma is limited. Reduced IL-10 spatial ranges result in a decreased total bacterial load with a slight increase in activated Mφs and a significant increase in apoptosis of resting Mφs. Increased IL-10 spatial ranges result in an increased total bacterial load with a decrease in activated Mφs and a decrease in apoptosis of resting Mφs. In contrast to the situation for TNF-α, these results suggest that a large spatial range of IL-10 inhibits apoptosis of resting Mφs in peripheral granuloma regions, but at the cost of limited Mφ activation. Yet, too small of an IL-10 spatial range cannot control TNF-α induced processes in the entire granuloma environment. Overall, our simulations demonstrate that spatial distributions of concentrations of TNF-α and IL-10 during *Mtb* infection are essential to maintaining effective levels of inflammation within regions containing bacteria, but limiting inflammatory processes in the outer regions of the granuloma that contain few, if any, bacteria.

### A Balance of TNF-α and IL-10 Concentrations Promotes an Environment that Contains Bacterial Growth with Minimal Tissue Damage

**Figure 7:**
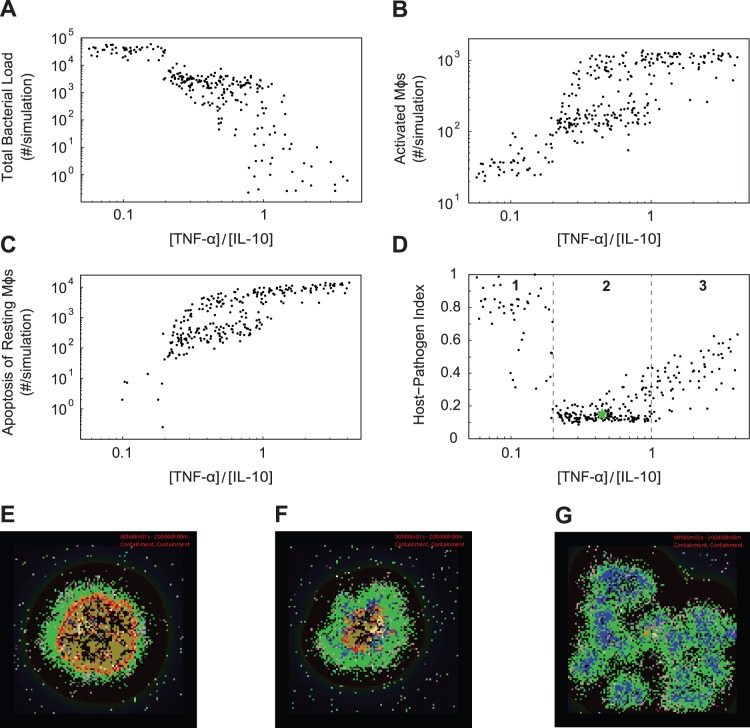
Altering the ratio of [TNF-α]/[IL-10] in a granuloma environment. Simulation results at 200 days post-infection showing the effects of altering the ratio of average tissue concentrations of TNF-α to IL-10 in the granuloma environment ([TNF-α]/[IL-10]). A total of 296 simulations (4 replications) were performed yielding various values of [TNF-α]/[IL-10]. Comparison of the ratio of concentrations of TNF-α to IL-10 with: A. B. C. Total bacterial load, number of activated Mφ, and number of apoptotic resting Mφ as a function of [TNF-α]/[IL-10]. D. Host-Pathogen Index (H.P.I), a metric that combines the three previous measures as a function of [TNF-α]/[IL-10]. The green star is the average simulation result for the baseline containment parameter set. E–G. Representative granuloma snapshots at 200 days post-infection for each of the regions (1, 2, and 3) defined in Figure 7D. For full length time-lapse simulations please see http://malthus.med.micro.umich.edu/lab/movies/TNF-IL10.

We have shown that three parameter groups are critical to controlling the average tissue concentrations of TNF-α and IL-10 and the subsequent responses necessary for granuloma function. Is there a balance of TNF-α and IL-10 concentrations that promotes strong immune cell activation, control of *Mtb* infection, and minimal host-induced tissue damage? To answer this question, we modulate concentrations of TNF-α and IL-10 available in a granuloma by simultaneously varying the synthesis of TNF-α mRNA by Mφs and the synthesis of IL-10 by activated Mφs. Each combination of parameters yields ratios of average tissue concentrations ([TNF-α]/[IL-10]), which we plot against representative model outputs of total bacterial load, number of activated Mφs, and apoptosis of resting Mφs at 200 days post-infection (Figure 7A–C).

When [TNF-α]/[IL-10] is less than ∼0.2 the immune response to *Mtb* in a granuloma is dulled, with low levels of activated Mφs and increased total bacterial loads, but with low apoptosis of resting Mφs. These granulomas are unable to contain the growth of *Mtb* and generally show larger lesion sizes and higher levels of B_ext_ (Figure 7E). Conversely, when [TNF-α]/[IL-10] is greater than ∼1.0, the immune response to *Mtb* infection in a granuloma is hyper-inflammatory, with high levels of apoptosis of resting Mφs, high levels of activated Mφs, and a low total bacterial load. This response causes significant tissue damage, while at the same time reducing bacterial loads and promoting clearance of bacteria. Granulomas that do achieve clearance of *Mtb* continue to activate Mφs, recruit T cells, and cause healthy cell death well beyond the removal of the pathogen, due to the lack of anti-inflammatory mediators (Figure 7G). When [TNF-α]/[IL-10] is between ∼0.2 and ∼1.0 simulations indicate that the granuloma response is balanced, with intermediate values of total bacterial load, low apoptosis of resting Mφs and sufficient numbers of activated Mφs (Figure 7F).

We calculate the H.P.I (defined in Materials and Methods) to provide a measure of overall granuloma function at different values of [TNF-α]/[IL-10] (Figure 7D). We devised this metric to incorporate the three measures just examined above - total bacterial load, apoptosis of resting Mφs (tissue damage), and activated Mφs (immune cell activation levels). Three general behaviors occur, and are indicated by regions 1, 2, and 3 in Figure 7D. Region 1, where the granuloma environment is dominated by IL-10, includes granulomas that display uncontrolled growth of *Mtb*, little to no Mφ activation and no apoptosis of resting Mφs (Figure 7E). In this region, the H.P.I is between ∼0.30–1.0. Region 2, where the granuloma environment is slightly IL-10 biased, includes granulomas that contain growth of *Mtb* with suitable levels of Mφ activation, low levels of apoptosis of resting Mφs, and a calculated H.P.I between ∼0.1–0.30 (Figure 7F). Region 3, with the TNF-α concentration dominating the granuloma environment, includes granulomas that contain or clear *Mtb* with uncontrolled Mφ activation, high levels of apoptosis of resting Mφs, and a calculated H.P.I. falling between ∼0.3–0.6 (Figure 7G). We see similar results for the balance of TNF-α and IL-10 in a granuloma at all time points beyond ∼75 days post-infection (data not shown). Our results demonstrate that a balance of concentrations of TNF-α and IL-10 promotes pathogen control while simultaneously preventing severe host tissue damage in a stable granuloma.

## Discussion

Granulomas are dynamic microenvironments consisting of an array of immune cells and cytokines. The time scale of persistent infection (typically years) coupled with the complexity of this environment, all contained within host lung tissue, limits experimental approaches to decipher many features of granulomas. To understand the opposing roles and impacts of TNF-α and IL-10 in granuloma formation and function across multiple spatial and temporal scales we use a systems biology approach. We extend our previous multi-scale hybrid agent-based model of *Mtb* infection by integrating TNF-α and IL-10 single-cell level receptor-ligand dynamics. We use our model to predict the impact of these dynamics on the longer-term and larger-scale cellular and tissue immune responses to *Mtb*.

We show that IL-10 is necessary to control activation levels and to prevent tissue damage in a granuloma. Simulations also predict that three groups of TNF-α and IL-10 parameters, representing processes relevant to cytokine synthesis, signaling, and spatial distribution, control concentrations of TNF-α and IL-10 in a granuloma environment and eventually determine infection outcome, at the tissue scale, over the long-term (Figure 5). We demonstrate that each parameter group is balancing a trade-off between host-induced tissue damage and bactericidal processes through various TNF-α and IL-10 mechanisms.

Spatial localization of TNF-α and IL-10 is important in focusing bactericidal and inflammatory activities in a granuloma (Figure 6D and Figure S3 in Appendix S4). In the core of the granuloma (where infected cells reside) bactericidal and inflammatory processes are necessary, while in the peripheral regions of the granuloma (where non-infected cells reside) cell death must be prevented through regulatory mechanisms. A balance of resting and infected Mφ activation with restricted apoptosis of uninfected Mφs is needed to contain bacterial growth with limited tissue damage. Both experimental and computational evidence point to the ability of immune cells to respond to different concentrations (across orders of magnitude) of TNF-α, via either NFκB activation or caspase-induced apoptosis, and IL-10, via anti-inflammatory or developmental transcription factors [13], [69], [80], [111]. Together with earlier studies on granuloma formation, this suggests the spatial organization of immune cells within a granuloma, where infected Mφs and *Mtb* reside in the center of the structure, is ideal for optimal control of bacteria [9], [80], [112].

We demonstrate for the first time that a balance of TNF-α and IL-10 concentrations is essential to mediate between *Mtb* infection control and prevention of host-induced tissue damage (Figure 7). Our results predict that granulomas with biased anti-inflammatory environments, having higher average concentrations of IL-10 than TNF-α, promote containment of bacteria and prevention of host tissue damage instead of bacterial clearance with high levels of healthy tissue damage. Shaler et al. [53] found that granuloma cells adopted a selectively suppressive phenotype when compared to airway lumen cells and proposed that granulomas may be immunosuppressive in nature. In contrast with the classical idea that granulomas function to wall off bacteria from its surroundings, granulomas may instead function as a niche that allows the survival of bacteria [11], [53]. What is still not understood is whether the nature of the granuloma environment arises due to the host-response or due to an immune-evasive mechanism of *Mtb* in order to support bacterial persistence, since hyper-virulent strains of *Mtb* can induce increased IL-10 production from macrophages [113]. A biased anti-inflammatory granuloma environment may have evolved over many generations from continued evolution of the host immune response and *Mtb* [20], [22]. During long-term containment, bacterial growth is restricted but are able persist in the host almost indefinitely, while the host shows no adverse signs of infection with limited host tissue damage. Thus, an anti-inflammatory biased granuloma that promotes containment may be the best outcome for both the host and the bacteria once infection has persisted past the initial stages of the immune response.

The balance of TNF-α and IL-10 concentrations in a granuloma presents a possible new avenue for treatment strategies. Granulomas that are ‘out of balance’ may need addition of antibodies or exogenous cytokines in order to shift from poorer outcomes and towards containment outcomes.

The simulations analyzed here focused on relatively mature granulomas. Strategies to treat early developing and less mature granulomas may differ, although the likelihood of detection and of infection at such an early stage (within weeks of infection) in a clinical setting is small. Anti-IL-10 and anti-IL-10R antibodies used *in-vivo* in the context of *Mtb* infection can result in increased bacterial control [22], [54]. Conversely, transgenic mice that overexpress IL-10 are more susceptible to *Mtb* infection and have an increased chance of reactivation [55]. However, since containment of bacteria appears to be an optimal outcome for both the host and the pathogen, it is still unclear how to treat these granulomas. We also note that there are other cytokines and immune cells, for example TGF-β and neutrophils, that may influence the immune response to *Mtb* [4]. Future studies could incorporate the dynamics of additional cytokines and immune cell types into an ABM to determine the effects of this complex milieu of cytokines interacting during *Mtb* infection.

Our modeling approach in this work represents a critical step towards fully understanding the roles of TNF-α and IL-10 and their effects on long-term *Mtb* infection outcome. In addition, the hybrid agent-based model platform we developed will allow us to rapidly explore new treatment strategies (such as immunomodulation) to affect the immune response to *Mtb*, narrowing the large design space for future experiments.

## Supporting Information

Appendix S1 **Agent-Based Model Rules and Interactions (GranSim).**
(DOC)

Appendix S2 **Molecular Scale Models.**
(DOC)

Appendix S3 **Model Parameters.**
(DOC)

Appendix S4 **Supplemental Data and Figures.**
(DOCX)
