# Supplementary material for: Multi-Scale Modeling Predicts a Balance of Tumor Necrosis Factor-α and Interleukin-10 Controls the Granuloma Environment during Mycobacterium tuberculosis Infection
Source: PLoS One. 2013 Jul 15;8(7):e68680. doi: 10.1371/journal.pone.0068680 (PMC3711807; doi:10.1371/journal.pone.0068680)
Supplement: Appendix S1 — Agent-Based Model Rules and Interactions (GranSim). (DOC) [file pone.0068680.s001.doc]

**Appendix S1 – Agent-Based Model Rules and Interactions (GranSim)**

The overall structure of the agent-based model (ABM) for the immune response to *Mtb* infection in the lung is presented below (Figure S1 in Appendix S1). Figure 2 in the main text indicates how individual models exist separately and how they are linked. Here, we describe the cellular and tissue scale model termed GranSim.

**Overall Structure of GranSim**

GRSim was developed based on four considerations: an environment, agents (immune cells), ABM rules that govern the agents and their interactions, and the time-step (Δt) used to update events. The environment represents a two-dimensional section of lung parenchyma as a 100 × 100 square lattice that simulates an area of 2 mm × 2 mm. Each grid micro-compartment is thus scaled to the approximate size of a single human macrophage, 20 μm in diameter. Discrete agents (macrophages and T cells) are recruited from specific micro-compartments on the lattice that represent vascular sources. Cells move on the lattice and interact with each other and the environment based on the ABM rules that are defined based on known biological activities. Due to the size difference between macrophages and T cells, up to two T cells are permitted to enter the same micro-compartment (with probability T_moveT_). A T cell may also move into the same micro-compartment as a macrophage (with probability T_moveM_). This model of cell spacing is a compromise between a realistic spatial representation and computational tractability and is consistent with observations on macrophage and T cell dynamics during development of mycobacterial granulomas that show granuloma-associated T cells squeeze through cell junctions created by a dense macrophage network [3].

Extracellular *Mtb* and soluble molecules, including chemokines (CCL2, CCL5 and CXCL9/10/11), soluble TNF-α (sTNF), shed TNFR2, and soluble IL-10, are simulated as continuous entities that can reside anywhere on the lattice. Extracellular Mtb grow in each micro-compartment. Soluble molecules diffuse and degrade among micro-compartments (See Appendix S2). Caseation represents inflammation of and damage to the lung parenchyma from macrophage cell death. In the ABM, caseation is defined to occur when a specific number (N_caseum_) of infected or activated macrophages die in a micro-compartment. When a micro-compartment becomes caseated, any T cell present in the micro-compartment is killed and no further cells are permitted to enter the micro-compartment.

There are two major types of discrete agents in the model, macrophages and T cells. Macrophages are either resting (M_r_, uninfected), infected (M_i_; have taken up *Mtb*), chronically infected (M_ci_; are unable to clear their intracellular *Mtb* due to a high bacterial load), or activated (M_a_; can effectively kill bacteria). Three distinct T cell classes based on their functions are modeled. The T_γ_ class represents CD4 and CD8 pro-inflammatory T cells; T_c_ class represents cytotoxic T cells (CTLs); and T_r_ class represents regulatory T cells including FOXP3+ and FOXP3- cells.

Cell-cell interactions are governed by ABM rules that are updated within every ABM time-step of dt_A_ = 10 min and will be discussed in the next section. Diffusion and degradation of soluble molecules on the lattice and secretion of chemokines from individual cells occurs is solved within each ABM time-step at a time step of dt_D_ = 30 seconds. TNF-α/TNFR and IL-10/IL-10R dynamics at the single-cell level are updated within the diffusion time-step with a shorter time-step of dt_M_ = 6 seconds. Molecular scale events are described in detail in Appendix S2. Thus, each single-cell event is updated 5 times within each diffusion time-step while the diffusion, degradation, and secretion events are updated 20 times with each ABM time-step. The overall algorithm of the simulation takes the form outlined in Figure S1 in Appendix S1 and will be presented in detail in the following sections.

**Figure S1. Overall outline of the hybrid multi-scale ABM granuloma simulations.** The boxes in gold are part of the tissue and cellular scale GRSim while the boxes in grey are part of the molecular scale model. dt_A_ is the ABM time-step. dt_D_ is the diffusion time step. dt_M_ is the single cell receptor ligand dynamics time-step.

**Grid Initialization**

A 100 × 100 two-dimensional grid is created. Periodic boundary conditions for cell movement and Dirichlet boundary conditions (zero outside grid perimeter) for molecular diffusion are used. 50 vascular source locations (N_source_) are distributed on the grid. 49 of the vascular sources are randomly distributed in 7 × 7 approximately equally sized partitions on the grid. One other micro-compartment is randomly selected from the whole grid as the last vascular source. Initial resting macrophages that represent resident alveolar macrophages are randomly placed on the grid (M_init_). One infected macrophage with one intracellular *Mtb* is placed at the center of the grid. This is consistent with estimations of the minimum infection dose of *Mtb* that range from a single bacterium upward [4].

**Cellular and Tissue Scale ABM Rules - Overview (GranSim)**

Cells move, become recruited to the site of infection, and respond to local conditions depending on their type and state according to rules that represent known biological activities *in vivo*. ABM rules that govern cell behaviors and interactions are as follows. Since chemokine single cell-level dynamics are not included in our model we discuss chemokine-related secretion and cellular recruitment in addition to cellular scale immunological details of the ABM in this section.

**Agent Movement**

*Macrophages*

Macrophages may stay in place or move in 8 possible directions on the grid based on CCL2 and CCL5 chemokine concentrations in their Moore neighborhood, the nine micro-compartments around the cell location including the micro-compartment occupied by the cell. Speed of movement only depends on the state of macrophages with the highest speed for M_r_ and the smallest speed (zero) for M_ci_. The differences among macrophage speeds are shown in the model by time intervals in which each macrophage attempts to move (t_moveMr_, t_moveMi_, t_moveMa_). There are minimum concentration thresholds and maximum saturating concentration thresholds (τ_chem_ and s_chem_) for the effect of each chemokine on cell movement. Chemokine concentrations below τ_chem_ or above s_chem_ do not have any extra effect on direction of movement. For simplicity we assume similar threshold values for all chemokines and cell types. Movement is random if chemokine concentrations in the Moore neighborhood are below τ_chem_ or above s_chem_. Otherwise, CCL2 and CCL5 concentrations in the Moore neighborhood determine a linear probability distribution for movement. We assume a bias for macrophage movement to the micro-compartment with the highest chemokine concentration. Hence, the highest chemokine concentration in the Moore neighborhood is multiplied by a factor 1.5 before calculation of movement probabilities. Movement is blocked by a caseous micro-compartment or macrophage presence and if blocked, no extra attempt for moving is made.

*T cells*

T cell movements are updated in time intervals of length t_MoveT_ that is determined by the speed of T cell migration *in vivo*. Movement of T_γ_ cells depends on CCL2, CCL5 and CXCL9/10/11 concentrations in the Moore neighborhood. T_c_ cells move based on CCL5 and CXCL9/10/11 concentrations and T_r_ cells move based on CCL5 concentrations. The details of T cell chemotactic movements are similar to macrophages as described above. T cell movement is blocked by caseation. However, T cell movement to a micro-compartment that contains one macrophage or one T cell is possible with reduced probabilities, T_moveM_ and T_moveT_, respectively.

**Cellular Recruitment**

We updated our previous cellular recruitment algorithm by adding chemokine- and cytokine-dependent recruitment rates of immune cells to the infection site. We recruit macrophages (M_r_) and T cells (T_γ_, T_c_, and T_r_) from vascular sources randomly distributed across the lung environment. The recruitment rate at each vascular source is dependent upon the concentrations of CCL2, CCL5, CXCL9, and TNF-α in the specified micro-compartment [5,6]. Recruitment at a vascular source that contains one macrophage or one T cell is subject to the same rules as movement with recruitment probabilities reduced by T_moveM_ and T_moveT_, respectively. The recruitment algorithm is shown in Figure S2 in Appendix S1 and described in detail below.

**Figure S2. Overall outline of the cellular recruitment algorithm.** The boxes in orange are defined in the appropriate agent section below. The stars represent a saved calculation from previous steps in the algorithm.

*Macrophages*

M_r_ are recruited every time-step from vascular sources based on available TNF-α and CCL2, and CCL5 concentrations at the specific vascular source, provided that the vascular source is not caseated nor blocked by a macrophage or two T cells. The probability of recruitment of M_r_ is given by:

$$P_{Mr}=M_{recMax}\left[\left(\frac{V_{Max}^{TNF}\left[TNF-\tau_{recMacTNF}\right]}{\left[TNF-\tau_{recMacTNF}\right]+h_{MacTNF}}\right)+\left(\frac{V_{Max}^{CCL2}\left[CCL2-\tau_{recMacCC}\right]}{\left[CCL2-\tau_{recMacCC}\right]+h_{MacCC}}\right)+\left(\frac{V_{Max}^{CCL5}\left[CCL5-\tau_{recMacCC}\right]}{\left[CCL5-\tau_{recMacCC}\right]+h_{MacCC}}\right)\right]$$

Along with the recruitment function the following threshold conditions must be met:

$$\left[CCL2\right]>\tau_{recMacCC}||\left[CCL5\right]>\tau_{recMacCC}||\left[TNF\right]>\tau_{recMacTNF}$$

*T cells*

Recruitment of T cells begins after a threshold of number of *Mtb* (N_MtbTcell_) is reached that represents the time required for activation of the adaptive immune response following *Mtb* infection. T_γ_ are recruited every time-step from vascular sources based on available TNF-α, CCL2, CCL5, and CXCL9/10/11 (written as only CXCL9 from here forth) concentrations at the specific vascular source, provided that the vascular source is not caseated nor blocked by a macrophage or two T cells. The probability of recruitment of T_γ_ is given by:

$$P_{T\gamma}=T_{recTgamMax}\left[\left(\frac{V_{Max}^{TNF}\left[TNF-\tau_{recTgamTNF}\right]}{\left[TNF-\tau_{recTgamTNF}\right]+h_{TgamTNF}}\right)+\left(\frac{V_{Max}^{CCL2}\left[CCL2-\tau_{recTagmCC}\right]}{\left[CCL2-\tau_{recTgamCC}\right]+h_{TagmCC}}\right)+\left(\frac{V_{Max}^{CCL5}\left[CCL5-\tau_{recTgamCC}\right]}{\left[CCL5-\tau_{recTgamCC}\right]+h_{TgamCC}}\right)+\left(\frac{V_{Max}^{CCL5}\left[CXCL9-\tau_{recTgamCC}\right]}{\left[CXCL9-\tau_{recTgamCC}\right]+h_{TgamCC}}\right)\right]$$

Along with the recruitment function the following threshold conditions must be met:

$$\left[CCL2\right]>\tau_{recMacCC}||\left[CCL5\right]>\tau_{recMacCC}||\left[CXCL9\right]>\tau_{recMacCC}||\left[TNF\right]>\tau_{recMacTNF}$$

T_c_ are recruited every time-step from vascular sources based on available TNF-α, CCL5, and CXCL9 concentrations at the specific vascular source, provided that the vascular source is not caseated nor blocked by a macrophage or two T cells. The probability of recruitment of T_c_ is given by:

$$P_{Tc}=T_{recTcytMax}\left[\left(\frac{V_{Max}^{TNF}\left[TNF-\tau_{recTcytTNF}\right]}{\left[TNF-\tau_{recTcytTNF}\right]+h_{TcytTNF}}\right)+\left(\frac{V_{Max}^{CCL5}\left[CCL5-\tau_{recTcytCC}\right]}{\left[CCL5-\tau_{recTcytCC}\right]+h_{TcytCC}}\right)+\left(\frac{V_{Max}^{CCL5}\left[CXCL9-\tau_{recTcytCC}\right]}{\left[CXCL9-\tau_{recTcytCC}\right]+h_{TcytCC}}\right)\right]$$

Along with the recruitment function the following threshold conditions must be met:

$$\left[CCL2\right]>\tau_{recMacCC}||\left[CXCL9\right]>\tau_{recMacCC}||\left[TNF\right]>\tau_{recMacTNF}$$

T_r_ are recruited every time-step from vascular sources based on available TNF-α and CCL5 concentrations at the specific vascular source, provided that the vascular source is not caseated nor blocked by a macrophage or two T cells. The probability of recruitment of T_r_ is given by:

$$P_{Tr}=T_{recTregMax}\left[\left(\frac{V_{Max}^{TNF}\left[TNF-\tau_{recTregTNF}\right]}{\left[TNF-\tau_{recTregTNF}\right]+h_{TregTNF}}\right)+\left(\frac{V_{Max}^{CCL5}\left[CCL5-\tau_{recTregCC}\right]}{\left[CCL5-\tau_{recTregCC}\right]+h_{TregCC}}\right)\right]$$

Along with the recruitment function the following threshold conditions must be met:

$$\left[CCL5\right]>\tau_{recMacCC}||\left[TNF\right]>\tau_{recMacTNF}$$

**Cell-Cell Interactions and State Transitions**

All cell-cell interactions and state transitions described below are updated every ABM time-step for all cells.

*Cell Death Due to Age*

All macrophages that are initially distributed or recruited on the grid are assigned a lifespan selected from a uniform distribution between zero and max_ageMac_. T cells are also assigned a lifespan randomly distributed between zero and max_ageTcell_. M_a_ has a shortened lifespan of max_ageActive_. At death, M_r_ and T cells are removed from the grid. At death, M_i_ and M_ci_ are removed from the grid and any intracellular *Mtb* (B_int_) is dispersed uniformly in the Moore neighborhood as extracellular bacteria (B_ext_). M_a_ death contributes to caseation of the micro-compartment.

*Rules for resting macrophages (M_r_)*

There is a chance of STAT-1 activation in a time-step as a result of interaction between a M_r_ and IFN-γ producing T_γ_ cells with a probability (nT_γ_ * P_STAT1_); where, nT_γ_ is the number of T_γ_ cells surrounding the M_r_ in the Moore neighborhood including the micro-compartment occupied by the M_r_. M_r_ can become NF-κB activated through TNF-α induced processes, which is discussed in Appendix S2. NF-κB activation can also occur if the B_ext_ in the Moore neighborhood exceeds B_actM_. STAT-1 and NF-κB activation last for the time interval t_STAT1_ and t_NFκB_ after which STAT-1 or NF-κB activation, respectively, is lost. M_r_ that are either STAT1 or NF-κB can be down-regulated by a T_r_ in which the M_r_ loses either STAT1 or NF-κB activation respectively. M_r_ is able to uptake or to kill B_ext_ that reside in the same micro-compartment. If the number of B_ext_ ≤ N_rk_, M_r_ kills them. Otherwise, it either kills N_rk_ of the B_ext_ with probability P_k_ or becomes infected (M_i_) after uptake of N_rk_ of the B_ext_ as its initial B_int_. M_r_ that are either STAT1 or NF-κB activated kill B_ext_ with a probability 2* P_k_ due to increased anti-microbial capacity. If both STAT1 and NF-κB are activated in a Mr and it is not already down-regulated by a T_r_, it becomes activated (M_a_). Following Tr down-regulation, M_r_ does nothing but moves for a fixed period of time t_regMac_. If the remaining lifespan of such an activated macrophage is greater than max_ageActive_, it will be shortened to max_ageActive_.

*Rules for infected macrophages (M_i_)*

B_int_ replicates in M_i_ every ABM time-step according to the following equation:

(Eq. 1)

M_i_ is able to uptake but not kill B_ext_ from its micro-compartment with a probability (P_uptakeMi_) that is computed as a function of B_int_ as follows:

$P_{uptakeMi}=(N_{c}-B_{\mathrm{int}})/100$ (Eq. 2)

M_i_ takes up N_rk_ of extracellular bacteria if B_ext_ > N_rk_. Otherwise, it takes up all extracellular bacteria that are available in the micro-compartment. If the number of B_int_ exceeds a threshold N_c_, the M_i_ becomes chronically infected (M_ci_). There is a chance of STAT-1 activation in a time-step as a result of interaction between a M_i_ and IFN-γ producing T_γ_ cells with a probability (nT_γ_ * P_STAT1_); where, nT_γ_ is the number of T_γ_ cells surrounding the M_r_ in the Moore neighborhood including the micro-compartment occupied by the M_r_. M_i_ can become NF-κB activated through TNF-α induced processes, which is discussed in Appendix S2. NF-κB activation can also occur if the B_ext_ in the Moore neighborhood exceeds B_actM_. STAT-1 and NF-κB activation last for the time interval t_STAT1_ and t_NFκB_ after which STAT-1 or NF-κB activation, respectively, is lost. M_i_ that are either STAT1 or NF-κB can be down-regulated by a T_r_ in which the M_i_ loses either STAT1 or NF-κB activation respectively. Following T_r_ down-regulation, M_i_ does nothing but moves for a fixed period of time t_regMac_, but continues to secrete chemokines. If both STAT1 and NF-κB are activated in a M_i_ and it is not already down-regulated by a T_r_, it becomes activated (M_a_). If the remaining lifespan of such an activated macrophage is greater than max_ageActive_, it will be shortened to max_ageActive_.

*Rules for chronically infected macrophages (M_ci_)*

B_int_ replicates in M_ci_ every time-step according to Eqn. 1. If the B_int_ exceeds a threshold (N_burst_), the M_ci_ bursts and its B_int_ are evenly distributed to the Moore neighborhood surrounding the M_ci_. M_ci_ bursting contributes to caseation of the micro-compartment. M_ci_ are always NF-κB activated and cannot become STAT1 activated. M_ci_ cannot be down-regulated by a T_r_.

*Rules for activated macrophages (M_a_)*

M_a_ is capable of effectively killing B_ext_. Each time-step, Ma kills N_ak_ of the B_ext_ in its micro-compartment. M_a_ that transitions from M_i_ kill B_int_ at the same rate that it kills B_ext_ each time-step. M_a_ can be down-regulated by a T_r_ in which the M_a_ loses both STAT1 and NF-κB activation. Following T_r_ down-regulation, M_a_ does nothing but moves for a fixed period of time t_regMac_ and subsequently transitions back to M_r_ after its down-regulated state.

*Rules for cytotoxic T cells (T_c_)*

If T_c_ is not already down-regulated by a T_r_ and there is a M_i_ or M_ci_ present in its Moore neighborhood there is a chance of perforin/granulysin-mediated killing of M_i_ or M_ci_ with probability P_cytKill_. If there are more than one M_i_ or M_ci_ in the Moore neighborhood one is chosen at random and the chance of perforin/granulysin-mediated killing of M_i_ or M_ci_ is given by the probability P_cytKill_. M_i_ killing by a T_c_ kills all B_int_ and contributes to caseation of the micro-compartment. In the case of M_ci_ killing, the B_int_ are killed with probability P_cytKillClean_. Otherwise, half of the B_int_ will be uniformly distributed in the Moore neighborhood. M_ci_ killing by T_c_ also contributes to caseation of the micro-compartment. When down-regulated, T_c_ cells lose their cytotoxic capabilities for a fixed period of time t_regTcyt_.

*Rules for pro-inflammatory T cells (T_γ_)*

If T_γ_ is not already down-regulated by a T_r_ and there is a M_i_ or M_ci_ present in its Moore neighborhood there is a chance of Fas/FasL-induced apoptosis of M_i_ or M_ci_ with probability P_apop/Fas_. If there are more than one M_i_ or M_ci_ in the Moore neighborhood one is chosen at random and the chance of Fas/FasL-induced apoptosis of M_i_ or M_ci_ is given by the probability P_apop/Fas_. As a result of apoptosis, half of the B_int_ in M_i_ or M_ci_ will be killed and the other half will be equally distributed in the Moore neighborhood as B_ext_. When down-regulated, T_γ_ cells lose their apoptotic capabilities for a fixed period of time t_regTgam_.

*Rules for regulatory T cells (T_r_)*

T_r_ suppresses or down-regulates the action of T cells and macrophages through non interleukin-10 mechanisms (CTLA-4, TGF-β, etc.), which are still poorly understood [7–9]. Thus, the probability of alternative suppressive functions of T_r_ occurring is linearly dependent upon the following ratio (Eq. 3), which coarsely simulates the mechanisms of other regulatory mechanisms that are not the focus of this work.

$BoundRatio=\frac{[TNF\cdot TNFR1]}{[TNF\cdot TNFR1]+[IL10\cdot IL10R]}$ (Eq. 3)

T_r_ here down-regulates all cells (macrophages, T_c_ and T_γ_) in its Moore neighborhood. Down-regulated states last for t_regMac_, t_regTgam_ and t_regTcyt_ for macrophages, T_c_ and T_γ_ cells, respectively. T_r_ down-regulation for each cell type is explained in sections that describe ABM rules for that specific cell type.

**Extracellular *Mtb* Growth**

Growth of extracellular *Mtb* (B_ext_) in all micro-compartments is calculated based on the following equation:

(Eq. 4)

**Chemokine Secretion**

M_i_, M_ci_, M_a_, NF-κB activated M_r_, and NF-κB activated M_i_ are able to secrete chemokines, provided that they are not down-regulated by T_r_. The rates of chemokine secretion for different cell types are as follows. M_ci_, M_a_ and NF-κB activated M_i_ are able to secrete chemokines with full secretion rates (r_CCL2_, r_CCL5_, and r_CXCL9_) as listed in Table S3 in Appendix S3. NF-κB activated M_r_ and M_i_ cells that are not NF-κB activated secrete chemokines with half-full secretion rates (0.5 × r_CCL2_, 0.5 × r_CCL5_, and 0.5 × r_CXCL9_). Caseated micro-compartments also secrete attractants that attract immune cells. For simplicity, we use quarter-full rates of chemokine secretion to simulate the effect of such attractants (0.25 × r_CCL2_, 0.25 × r_CCL5_, and 0.25 × r_CXCL9_). Chemokine secretions to the micro-compartments on the grid are updated in time intervals of dt_D_. Secretion of TNF and IL-10 is discussed in Appendix S2.

**Visualization and Cross-Platform Capabilities**

We have revised the user interface for performing our modeling studies so that we can easily visualize and track different aspects of the granuloma, including the structure and molecular concentration gradients, as it forms and is maintained. In order to satisfy the cross-platform requirement, we make use of the Qt framework. Qt is a C++ framework for developing cross-platform applications with a graphical user-interface (open-source, distributed under GPL – available at qt.digia.com).

**References**

1. Ray JCJ, Flynn JL, Kirschner DE (2009) Synergy between individual TNF-dependent functions determines granuloma performance for controlling Mycobacterium tuberculosis infection. Journal of immunology (Baltimore, Md : 1950) 182: 3706–3717. doi:10.4049/jimmunol.0802297.

2. Fallahi-Sichani M, El-Kebir M, Marino S, Kirschner DE, Linderman JJ (2011) Multiscale computational modeling reveals a critical role for TNF-α receptor 1 dynamics in tuberculosis granuloma formation. Journal of immunology (Baltimore, Md : 1950) 186: 3472–3483. doi:10.4049/jimmunol.1003299.

3. Egen JG, Rothfuchs AG, Feng CG, Winter N, Sher A, et al. (2008) Macrophage and T cell dynamics during the development and disintegration of mycobacterial granulomas. Immunity 28: 271–284. doi:10.1016/j.immuni.2007.12.010.

4. Ford CB, Lin PL, Chase MR, Shah RR, Iartchouk O, et al. (2011) Use of whole genome sequencing to estimate the mutation rate of Mycobacterium tuberculosis during latent infection. Nature genetics 43: 482–486. doi:10.1038/ng.811.

5. Vesosky B, Rottinghaus EK, Stromberg P, Turner J, Beamer G (2010) CCL5 participates in early protection against Mycobacterium tuberculosis. Journal of leukocyte biology 87: 1153–1165. doi:10.1189/jlb.1109742.

6. Chabot V, Reverdiau P, Iochmann S, Rico A, Sénécal D, et al. (2006) CCL5-enhanced human immature dendritic cell migration through the basement membrane in vitro depends on matrix metalloproteinase-9. Journal of leukocyte biology 79: 767–778. doi:10.1189/jlb.0804464.

7. Tang Q, Bluestone J a (2008) The Foxp3+ regulatory T cell: a jack of all trades, master of regulation. Nature immunology 9: 239–244. doi:10.1038/ni1572.

8. Rubtsov YP, Rasmussen JP, Chi EY, Fontenot J, Castelli L, et al. (2008) Regulatory T cell-derived interleukin-10 limits inflammation at environmental interfaces. Immunity 28: 546–558. doi:10.1016/j.immuni.2008.02.017.

9. Shevach EM (2009) Mechanisms of foxp3+ T regulatory cell-mediated suppression. Immunity 30: 636–645. doi:10.1016/j.immuni.2009.04.010.
