# Supplementary material for: Multi-Scale Modeling Predicts a Balance of Tumor Necrosis Factor-α and Interleukin-10 Controls the Granuloma Environment during Mycobacterium tuberculosis Infection
Source: PLoS One. 2013 Jul 15;8(7):e68680. doi: 10.1371/journal.pone.0068680 (PMC3711807; doi:10.1371/journal.pone.0068680)
Supplement: Appendix S2 — Molecular Scale Models. (DOC) [file pone.0068680.s002.doc]

**Appendix S2 – Molecular Scale Models**

The overall structure of the computational model of the immune response to *Mtb* infection in the lung is presented in Appendix S1. Figure 2 in the Manuscript indicates how these models exist separately and how they are linked. All parameter definitions and values are listed in Appendix S3. The overall algorithm of the simulation takes the form outlined in Figure S1 in Appendix S1 and the molecular scale will be presented in detail in this section. The solution of molecular scale models of diffusion, degradation, and TNF-α and IL-10 single-cell level receptor-ligand dynamics will be discussed in this Appendix.

**Diffusion of Soluble Molecules**

The two-dimensional partial differential equation (PDE) for diffusion of CCL2, CCL5, CXCL9, TNF-α, IL-10, and shed bound TNFR2 is given by the following equation.

$\frac{\partial C}{\partial t}=D\left(\frac{\partial^{2}C}{\partial x^{2}}+\frac{\partial^{2}C}{\partial y^{2}}\right)$ (Eq. 1)

C is the concentration of diffusing molecule that changes with time (t) in the x and y directions and D is the diffusion coefficient for the molecule in the tissue environment. This equation can be implemented numerically on the grid by using the alternate-direction explicit finite difference discrete-time discrete-space approximation scheme [3]. Let *u*_i, j_ and *v*_i, j_ be finite difference solutions of the transient diffusion PDE. Define i as the lattice parameter for the x-direction, j as the lattice parameter for y-direction, n as the current time point, Δx as the x-direction grid size, and Δy as the y-direction grid size. In the case of *u* the iteration proceeds forward in the i, j direction thus any values of *u* at time (n+1) will be known. For the case of *v* the iteration proceeds in the decreasing i, j direction (the iteration starts at the end of the grid and works backwards) therefore all values of *v* at time (n+1) will be known.

$\frac{u_{i,j}^{n+1}-u_{i,j}^{n}}{\delta t}=\frac{u_{i+1,j}^{n}-u_{i,j}^{n}-u_{i,j}^{n+1}+u_{i-1,j}^{n+1}}{\delta x^{2}}+\frac{u_{i,j+1}^{n}-u_{i,j}^{n}-u_{i,j}^{n+1}+u_{i,j-1}^{n+1}}{\delta y^{2}}$ (Eq. 2)

(Eq. 3)

Thus, the concentration at a specific time point is given by the arithmetic average of u and v.

$C_{i,j}^{n+1}=\frac{u_{i,j}^{n+1}+v_{i,j}^{n+1}}{2}$ (Eq. 4)

This method is unconditionally stable, allowing us to choose a dt_D_ = 30 seconds for reasonable numerical accuracy and computational efficiency.

**Degradation of Soluble Molecules**

Degradation of soluble CCL2, CCL5, CXCL9, TNF-α, IL-10, and shed bound TNFR2 is described by:

$\frac{\partial C}{\partial t}=-k_{\deg}C$ (Eq. 5)

where k_deg_ is the degradation rate constant for the specific molecule in the tissue environment. In order to increase the accuracy of the solution degradation and prevent unnecessary computational burden, we calculate degradation using the analytical solution of Eqn. 5.

(Eq. 5)

This is calculated with the same solver time step as the diffusion equation, dt_D_.

**Single-Cell Level Receptor-Ligand Dynamics**

We use ordinary differential equations (ODEs) to describe the TNF/TNFR and IL-10/IL-10R receptor-ligand dynamics occurring at the single-cell level. TNF/TNFR and IL-10/IL-10R ODEs are solved for each individual cell on the grid using the time-step dt_M_ and the 4^th^ order Runge-Kutta numerical method. Soluble molecules in the model (sTNF, sTNF/TNFR2_shed_, and IL-10) are expressed as volumetric concentration units (e.g. M), whereas cell-associated species are expressed as numbers of molecules per cell. Thus, when a membrane-bound molecule releases to the extracellular space (i.e. the micro-compartment occupied by the cell), or when a soluble molecule binds to the cell membrane, a scaling factor (ρ/Nav) is required as indicated in Table S2 in Appendix S2, where ρ is the cell density in the micro-compartment and can be computed as dx^3^ assuming that each micro-compartment is a cube of side dx.

*Single-Cell Level IL-10/IL-10R Equations*

The binding interactions and reactions controlling IL-10/IL-10R dynamics at the single-cell level regardless of the cell type are schematically illustrated in the main text (Figure 2). IL-10 is synthesized by IL-10 producing cells (M_i_, M_ci_, M_a_, and T_r_), if not down-regulated by T_r_ cells, and is released directly into the extracellular environment [4–12]. IL-10 exists in the extracellular space as a non-covalently bonded dimer where it can bind to cell-surface IL-10R1 and IL-10R2 [13,14]. Signaling occurs through association of bound IL-10R1 with the IL-10R2 subunit [5]. IL-10R1 is the high affinity receptor compared to IL-10R2, which mainly exists as a signaling subunit to bound IL-10R1. For simplicity, we include only a general IL-10R that represents both IL-10R1 and IL-10R2 [15,16]. IL-10R is synthesized by the cell and is removed from the membrane by turnover [17]. Bound IL-10R can be internalized where it can be degraded or recycled to the surface [18]. The processes of degradation and recycling are not explicitly modeled since tracking internalized bound IL10R adds unnecessary complexity to the ODEs. We lump these processes into IL10R turnover and synthesis for simplicity. We modeled these molecular processes based on mass action kinetics as shown in Table S1 in Appendix S2; model equations are listed in Table S2 in Appendix S2; definitions and values of the rate constants are given in Table S4 in Appendix S3.

The rates of IL-10 synthesis and IL-10R synthesis (k_Synth_ and V_r_) are cell type/state-specific as indicated in Table S4 in Appendix S3, but other rate constant values are common between all cells. In the multi-scale model described in this work, the rates of IL-10 synthesis for different cell types are as follows. M_i_ are able to synthesize IL-10 at the full rate k_synthMacInf_, while M_ci_ make IL-10 at 1.5* k_synthMacInf_ shown in Table S4 in Appendix S3. M_a_ synthesize IL-10 at a basal rate of k_synthMacAct_ but the rate is dependent upon the bound TNFR1 concentration as shown in Table S2 in Appendix S2 below. T_r_ cells express IL-10 at the full rate k_synthTcell_, while T_γ_ and T_c_ do not synthesize IL-10 in our model.

*Single-Cell Level TNF/TNFR Ordinary Equations*

The binding interactions and reactions controlling TNF/TNFR dynamics at the single-cell level regardless of the cell type are adapted from [2] and are schematically illustrated in (Figure 2). TNF-α mRNA is transcribed by TNF-producing cells (M_i_, M_ci_, M_a_, NF-κB activated M_r_, T_γ_ and T_c_), if not down-regulated by T_r_ cells, and subsequently translated into its membrane bound form, mTNF. mTNF is then processed and released as a soluble form (sTNF) into extracellular spaces. This processing occurs via a cell-associated metalloproteinase called TACE. Two types of TNF receptors (TNFR1 and TNFR2) are synthesized and expressed on the cell surface as free receptors. Soluble TNF (sTNF) reversibly binds to TNFRs on the cell membrane. sTNF-bound cell surface TNFR1 internalizes and sTNF-bound cell surface TNFR2 may undergo internalization or shedding into extracellular spaces [8]. Internalized receptors may degrade or recycle to the cell membrane where they can re-bind to sTNF [9]. Ligand-free TNFRs also turn over (internalize) [9,10]. Intact sTNF may dissociate from the shed sTNF/TNFR2 complex in the extracellular space [11]. We modeled these molecular processes based on mass action kinetics as shown in Table S1 in Appendix S2; model equations are listed in Table S2 in Appendix S2; definitions and values of the rate constants are given in Table S4 in Appendix S3.

The rates of TNF-α mRNA synthesis and release from the cell membrane and TNFR synthesis (k_mRNA_, k_Synth_, k_TACE_, V_r1_ and V_r2_) are cell type/state-specific as indicated in Table S4 in Appendix S3, but other rate constant values are common between all cells. In the multi-scale model described in this work, the rates of TNF-α mRNA synthesis for different cell types are as follows. M_ci_, M_a_ and NF-κB activated M_i_ are able to synthesize mTNF with a full rate (k_mRNA_ = kmRNA_Mac and k_synth_ = k_synthMac_) as shown in Table 4 in Appendix S3. NF-κB activated M_r_ and non-NF-κB activated M_i_ express TNF-α mRNA with a half-full rate (k_mRNA_ = 0.5*kmRNA_Mac and k_synth_ = 0.5*k_synthMac_). T_γ_ cells express TNF-α mRNA at a rate of kmRNA_Tcell and k_synth_ = k_synthTcell_, while T_c_ cells express TNF-α mRNA at a rate of k_mRNA_ = 0.1*kmRNA_Tcell and k_synth_ = 0.1*k_synthTcell_. T_r_ cells do not express TNF. TACE activity is also assumed to be cell type-dependent as shown in Table S4 in Appendix S3.

*Linking TNF/TNFR and IL-10/IL-10R Equations*

TNF-α and IL-10 receptor-ligand dynamics are linked in two ways as shown in Figure 2 in the Manuscript. Bound IL-10R inhibits TNF-α mRNA transcription while bound TNFR1 can induce synthesis of IL-10 in M_a_. Inhibition of TNF-α mRNA transcription shows rapid switch-like behavior, thus we modeled these processes with a three-parameter logistic function [19].

$k_{mRNA-Mod}=k_{mRNA}\left(\beta+\frac{1-\beta}{1+e^{\frac{[IL10\cdot IL10R]-\gamma_{I}}{\delta_{I}}}}\right)$ (Eq. 6)

We captured the ability of bound TNFR1 to induce synthesis of IL-10 in M_a_ with Michaelis-Menton type kinetics, which roughly approximates the mechanisms influencing the plasticity of M_a_ to produce IL-10 at lower (classical M_a_) or higher (alternative M_a_) rates [20–24].

$k_{synthMacAct-Mod}=k_{synthMacAct}\left(\frac{[sTNF\cdot TNFR1]}{[sTNF\cdot TNFR1]+h_{synthMacAct}}\right)$ (Eq. 7)

**Linking the Molecular Scale to GranSim**

The molecular scale sub-models of TNF-α are linked to GRSim through NFκB activation of macrophages, caspase induced cell apoptosis, and cellular recruitment. We describe TNF-α induced NFκB activation of each macrophage and TNF-α induced apoptosis of cells as Poisson processes with a probability of occurrence determined by a rate constant, threshold value, and a saturation value (see Table S4 in Appendix S3 for parameter definitions and values) [2]. TNF-α induced NFκB activation of macrophages is dependent on the concentration of bound TNFR1 per cell (Eq. 8), while TNF-α induced apoptosis is dependent on the concentration of internalized bound TNFR1 per cell (Eq. 9).

$P_{NFkB}=\left\{\begin{matrix} 1-e^{-k_{NFkB}([sTNF-TNFR1]-\tau_{NFkB})\Delta t} & [sTNF-TNFR1]\geq\tau_{NFkB} \\ 0 & [sTNF-TNFR1]<\tau_{NFkB} \end{matrix}\right\}$ (Eq. 8)

$P_{apop}=\left\{\begin{matrix} 1-e^{-k_{apop}([sTNF-TNFR1_{i}]-\tau_{apop})\Delta t} & [sTNF-TNFR1_{i}]\geq\tau_{apop} \\ 0 & [sTNF-TNFR1_{i}]<\tau_{apop} \end{matrix}\right\}$ (Eq. 9)

NF-κB activation is checked once for all M_r_ and M_i_ within each ABM time-step (dt_A_). TNF-induced apoptosis is checked once for all cells on the grid within each ABM time-step (dt_A_). The molecular scale IL-10 sub-models are linked to GRSim through chemokine down regulation and compensation of alternative suppressive functions. IL-10 inhibits the production of chemokines by macrophages; we use a simple threshold relationship, wherein the synthesis of chemokines is reduced in half once the number of bound IL-10R is above a specified value [25–28]. The probability of alternative suppressive functions of T_r_ occurring is linearly dependent on the ratio of bound TNFR1 to bound IL-10R, which coarsely simulates the mechanisms of other regulatory mechanisms that are not the focus of this work [6,29–33].

**Table S1.** Molecular Scale Single-Cell TNF/TNFR and IL10/IL10R Equations – Model Reactions and Rates (*v*_i_).

**Table S2.** Molecular Scale Single-Cell TNF/TNFR and IL10/IL10R Equations.

**References**

1. Ray JCJ, Flynn JL, Kirschner DE (2009) Synergy between individual TNF-dependent functions determines granuloma performance for controlling Mycobacterium tuberculosis infection. Journal of immunology (Baltimore, Md : 1950) 182: 3706–3717. doi:10.4049/jimmunol.0802297.

2. Fallahi-Sichani M, El-Kebir M, Marino S, Kirschner DE, Linderman JJ (2011) Multiscale computational modeling reveals a critical role for TNF-α receptor 1 dynamics in tuberculosis granuloma formation. Journal of immunology (Baltimore, Md : 1950) 186: 3472–3483. doi:10.4049/jimmunol.1003299.

3. Barakat HZ, Clark JA (1966) On the Solution of the Diffusion Equations by Numerical Methods. Journal of Heat Transfer 88: 421–427.

4. Duell BL, Tan CK, Carey AJ, Wu F, Cripps AW, et al. (2012) Recent insights into microbial triggers of interleukin-10 production in the host and the impact on infectious disease pathogenesis. FEMS immunology and medical microbiology. doi:10.1111/j.1574-695X.2012.00931.x.

5. Moore KW, De Waal Malefyt R, Coffman RL, O’Garra A (2001) Interleukin-10 and the interleukin-10 receptor. Annual review of immunology 1: 683–765.

6. Rubtsov YP, Rasmussen JP, Chi EY, Fontenot J, Castelli L, et al. (2008) Regulatory T cell-derived interleukin-10 limits inflammation at environmental interfaces. Immunity 28: 546–558. doi:10.1016/j.immuni.2008.02.017.

7. Wu K, Koo J, Jiang X, Chen R, Cohen SN, et al. (2012) Improved control of tuberculosis and activation of macrophages in mice lacking protein kinase R. PloS one 7: e30512. doi:10.1371/journal.pone.0030512.

8. Giacomini E, Iona E, Ferroni L, Miettinen M, Fattorini L, et al. (2001) Infection of human macrophages and dendritic cells with Mycobacterium tuberculosis induces a differential cytokine gene expression that modulates T cell response. Journal of immunology (Baltimore, Md : 1950) 166: 7033–7041.

9. Yssel H, De Waal Malefyt R, Roncarolo MG, Abrams JS, Lahesmaa R, et al. (1992) IL-10 is produced by subsets of human CD4+ T cell clones and peripheral blood T cells. Journal of immunology (Baltimore, Md : 1950) 149: 2378–2384.

10. Meyaard L, Hovenkamp E, Otto SA, Miedema F (1996) IL-12-induced IL-10 production by human T cells as a negative feedback for IL-12-induced immune responses. Journal of immunology (Baltimore, Md : 1950) 156: 2776–2782.

11. Orme IM, Roberts AD, Griffin JP, Abrams JS (1993) Cytokine secretion by CD4 T lymphocytes acquired in response to Mycobacterium tuberculosis infection. Journal of immunology (Baltimore, Md : 1950) 151: 518–525.

12. Shaw TC, Thomas LH, Friedland JS (2000) Regulation of IL-10 secretion after phagocytosis of Mycobacterium tuberculosis by human monocytic cells. Cytokine 12: 483–486. doi:10.1006/cyto.1999.0586.

13. Tan JC, Indelicato SR, Narula SK, Zavodny PJ, Chou CC (1993) Characterization of interleukin-10 receptors on human and mouse cells. The Journal of biological chemistry 268: 21053–21059.

14. Weber-Nordt RM, Meraz M a, Schreiber RD (1994) Lipopolysaccharide-dependent induction of IL-10 receptor expression on murine fibroblasts. Journal of immunology (Baltimore, Md : 1950) 153: 3734–3744.

15. Figueiredo AS, Höfer T, Klotz C, Sers C, Hartmann S, et al. (2009) Modelling and simulating interleukin-10 production and regulation by macrophages after stimulation with an immunomodulator of parasitic nematodes. The FEBS journal 276: 3454–3469. doi:10.1111/j.1742-4658.2009.07068.x.

16. Moya C, Huang Z, Cheng P, Jayaraman A, Hahn J (2011) Investigation of IL-6 and IL-10 signalling via mathematical modelling. IET systems biology 5: 15. doi:10.1049/iet-syb.2009.0060.

17. Lauffenburger DA, Linderman JJ (1993) Receptors: Models For Binding, Trafficking, and Signaling New York: Oxford University Press.

18. Wei SH-Y, Ming-Lum A, Liu Y, Wallach D, Ong CJ, et al. (2006) Proteasome-mediated proteolysis of the interleukin-10 receptor is important for signal downregulation. Journal of interferon & cytokine research : the official journal of the International Society for Interferon and Cytokine Research 26: 281–290. doi:10.1089/jir.2006.26.281.

19. Smallie T, Ricchetti G, Horwood NJ, Feldmann M, Clark AR, et al. (2010) IL-10 inhibits transcription elongation of the human TNF gene in primary macrophages. The Journal of experimental medicine 207: 2081–2088. doi:10.1084/jem.20100414.

20. Flynn JL, Chan J, Lin PL (2011) Macrophages and control of granulomatous inflammation in tuberculosis. Mucosal immunology 4: 271–278. doi:10.1038/mi.2011.14.

21. Schreiber T, Ehlers S, Heitmann L, Rausch A, Mages J, et al. (2009) Autocrine IL-10 induces hallmarks of alternative activation in macrophages and suppresses antituberculosis effector mechanisms without compromising T cell immunity. Journal of immunology (Baltimore, Md : 1950) 183: 1301–1312. doi:10.4049/jimmunol.0803567.

22. Verreck F a W, De Boer T, Langenberg DML, Hoeve M a, Kramer M, et al. (2004) Human IL-23-producing type 1 macrophages promote but IL-10-producing type 2 macrophages subvert immunity to (myco)bacteria. Proceedings of the National Academy of Sciences of the United States of America 101: 4560–4565. doi:10.1073/pnas.0400983101.

23. Wanidworanun C, Strober W (1993) Predominant role of tumor necrosis factor-alpha in human monocyte IL-10 synthesis. Journal of immunology (Baltimore, Md : 1950) 151: 6853–6861.

24. Platzer C, Meisel C, Vogt K, Platzer M, Volk HD (1995) Up-regulation of monocytic IL-10 by tumor necrosis factor-alpha and cAMP elevating drugs. International immunology 7: 517–523.

25. Hosokawa Y, Hosokawa I, Ozaki K, Nakae H, Matsuo T (2009) Cytokines differentially regulate CXCL10 production by interferon-gamma-stimulated or tumor necrosis factor-alpha-stimulated human gingival fibroblasts. Journal of periodontal research 44: 225–231. doi:10.1111/j.1600-0765.2008.01124.x.

26. Ikeda T, Sato K, Kuwada N, Matsumura T, Yamashita T, et al. (2002) Interleukin-10 differently regulates monocyte chemoattractant protein-1 gene expression depending on the environment in a human monoblastic cell line, UG3. Journal of leukocyte biology 72: 1198–1205.

27. Cheeran MC-J, Hu S, Sheng WS, Peterson PK, Lokensgard JR (2003) CXCL10 production from cytomegalovirus-stimulated microglia is regulated by both human and viral interleukin-10. Journal of virology 77: 4502–4515. doi:10.1128/JVI.77.8.4502.

28. Marfaing-Koka a, Maravic M, Humbert M, Galanaud P, Emilie D (1996) Contrasting effects of IL-4, IL-10 and corticosteroids on RANTES production by human monocytes. International immunology 8: 1587–1594.

29. O’Garra A, Vieira P, Vieira P, Goldfeld AE (2004) IL-10–producing and naturally occurring CD4+ Tregs: limiting collateral damage. Journal of Clinical Investigation 114: 1–7. doi:10.1172/JCI200423215.1372.

30. Cyktor JC, Turner J (2011) IL-10 and Immunity Against Prokaryotic and Eukaryotic Intracellular Pathogens. Infection and immunity. doi:10.1128/IAI.00047-11.

31. Roncarolo MG, Battaglia M, Bacchetta R, Fleischhauer K, Levings MK (2006) Interleukin-10-secreting type 1 regulatory T cells in rodents and humans. Immunological Reviews 212: 28–50. doi:10.1111/j.0105-2896.2006.00420.x.

32. Tang Q, Bluestone J a (2008) The Foxp3+ regulatory T cell: a jack of all trades, master of regulation. Nature immunology 9: 239–244. doi:10.1038/ni1572.

33. Shevach EM (2009) Mechanisms of foxp3+ T regulatory cell-mediated suppression. Immunity 30: 636–645. doi:10.1016/j.immuni.2009.04.010.
