# Supplementary material for: Multi-Scale Modeling Predicts a Balance of Tumor Necrosis Factor-α and Interleukin-10 Controls the Granuloma Environment during Mycobacterium tuberculosis Infection
Source: PLoS One. 2013 Jul 15;8(7):e68680. doi: 10.1371/journal.pone.0068680 (PMC3711807; doi:10.1371/journal.pone.0068680)
Supplement: Appendix S3 — Model Parameters. (DOC) [file pone.0068680.s003.doc]

**Appendix S3 – Model Parameters**

**Table S3. Tissue and cellular scale parameters.**

| **Parameter** | **Parameter Description** | **Value^*^** |
| --- | --- | --- |
| **Grid/Simulation Related Parameters** | | |
| *t_diffusion_* | Time step for solving diffusion PDEs | 30 |
| *t_molecular_* | Time step for solving molecular scale ODEs | 6 |
| *N_source_* | Number of vascular sources | 50 |
| *N_caseum_* | Number of qualified cell deaths required for caseation | 15 |
| *K_be_* | Capacity of a micro-compartment for extracellular Mtb | 200 |
| **Chemokine Related Parameters** | | |
| *D_chem_* (cm^2^/s) | Diffusion coefficient of chemokines | 10^-8^-10^-7^ (5.2×10^-8^) |
| *k_degChem_*(s^-1^) | Chemokine degradation rate constant | 5x10^-4^-5x10^-3^ (1.58×10^-3^) |
| *τ_chem_* (molecules) | Minimum chemokine concentration threshold | 1-10 (2) |
| *s_chem_* (molecules) | Saturating chemokine concentration threshold | 10^3^-10^4^ (2000) |
| *I_50IL10_* (ng/mL) | IC_50_ for IL10 inhibition of chemokine production | 10-30 (20) |
| **Macrophage Related Parameters** | | |
| *M_init_* | Initial number of resident macrophages | 105 |
| *max_ageMac_* (day) | Maximum lifespan of macrophages | 100 |
| *max_ageActive_* (day) | Maximum lifespan of an active macrophage | 10 |
| *t_regMac_* (hours) | Macrophage inactivity time after down-regulation by T_reg_ | 24 |
| *t_moveMr_* (min) | Time interval for M_r_ movement | 20 |
| *t_moveMa_* (hour) | Time interval for M_a_ movement | 7.8 |
| *t_moveMi_* (hour) | Time interval for Mi movement | 24 |
| *r_CCL2_* (molecules/s) | Full secretion rate of CCL2 by macrophages | 4.14 |
| *r_CCL5_* (molecules/s) | Full secretion rate of CCL5 by macrophages | 4.14 |
| *r_CXCL9_* (molecules/s) | Full secretion rate of CXCL9/10/11 by macrophages | 8.28 |
| *N_rk_* | Number of extracellular Mtb engulfed by M_r_ or M_i_ | 1 |
| *P_k_* | Probability of M_r_ killing bacteria | 0.2-0.3 (0.269) |
| *B_actM_* | Number of extracellular Mtb activating NF-κB in a mac | 150-300 (239) |
| *N_c_* | Number of intracellular Mtb for M_i_→M_ci_ transition | 10 |
| *N_burst_* | Number of intracellular Mtb that leads to M_ci_ bursting | 20-30 (20) |
| *P_STAT1_* | Probability of STAT-1 activation in M_r_ or M_i_ | 0.001-0.15 (0.0917) |
| *N_ak_* | Number of extracellular Mtb killed by M_a_ each time-step | 2-4 (3) |
| *τ_recMacCC_* | Chemokine threshold for M_r_ recruitment | 0.065-0.65 (0.151) |
| *τ_recMacTNF_* | TNF threshold for M_r_ recruitment | 0.006-0.06 (0.014) |
| *h_MacCC_* | Half-saturation of chemokines for M_r_ recruitment | 1-10 (5) |
| *h_MacTNF_* | Half-saturation of TNF for M_r_ recruitment | 0.09-0.9 (0.5) |
| *M_recMax_* | Maximum recruitment probability for M_r_ recruitment | 0.07-0.11 (0.09) |
| *t_STAT1_* (hours) | Time interval for STAT1 activation of M_r_ or M_i_ | 25-58 (41) |
| *t_NFκB_* (min) | Time interval for NFκB activation of M_r_ or M_i_ | 10-500 (100) |
| **T Cell Related Parameters** | | |
| *N_MtbTcell_* (# of Mtb) | Number of Mtb required to begin T cell recruitment | 50 |
| *max_ageTcell_* (days) | Maximum lifespan of T cells | 3 |
| *T_moveM_* | Probability of T cell moving to a mac-containing location | 0.005-0.05 (0.027) |
| *T_moveT_* | Probability of T cell moving to a T cell-containing location | 0.01-0.12 (0.08) |
| *t_regTgam_* (hours) | T_γ_ inactivity time after down-regulation by T_reg_ | 6 |
| *P_Fas/FasL_* | Probability of Fas/FasL apoptosis by T_γ_ | 0.005-0.05 (0.0095) |
| *τ_recTgamCC_* | Chemokine threshold for T_γ_ recruitment | 0.065-0.65 (0.151) |
| *τ_recTgamTNF_* | TNF threshold for T_γ_ recruitment | 0.006-0.06 (0.014) |
| *h_TgamCC_* | Half-saturation of chemokines for T_γ_ recruitment | 0.5-5 (1.5) |
| *h_TgamTNF_* | Half-saturation of TNF for T_γ_ recruitment | 0.05-0.5 (0.3) |
| *T_recTgamMax_* | Maximum recruitment probability for T_γ_ recruitment | 0.03-0.11 (0.0713) |
| *t_regTcyt_* (hours) | T_c_ inactivity time after down-regulation by T_reg_ | 6 |
| *P_cytKill_* | Probability of T_c_ killing M_i_ or M_ci_ | 0.005-0.05 (0.0098) |
| *P_cytKillClean_* | Probability of T_c_ killing all intracellular Mtb by killing M_ci_ | 0.75 |
| *τ_recTcytCC_* | Chemokine threshold for T_c_ recruitment | 0.08-0.85 (0.3775) |
| *τ_recTcytTNF_* | TNF threshold for T_c_ recruitment | 0.006-0.06 (0.014) |
| *h_TcytCC_* | Half-saturation of chemokines for T_c_ recruitment | 0.5-5 (1.5) |
| *h_TcytTNF_* | Half-saturation of TNF for T_c_ recruitment | 0.05-0.5 (0.3) |
| *T_recTcytMax_* | Maximum recruitment probability for T_c_ recruitment | 0.01-0.09 (0.0505) |
| *d_slopeTreg_* | Slope of deactivation probability function for T_reg_ | 0.75-1.0 (0.8905) |
| *d_minTreg_* | Minimum of deactivation probability function for T_reg_ | 0.0001-0.001 (0.0009) |
| *τ_recTregCC_* | Chemokine threshold for T_reg_ recruitment | 0.02-0.12 (0.0755) |
| *τ_recTregTNF_* | TNF threshold for T_reg_ recruitment | 0.006-0.06 (0.014) |
| *h_TregCC_* | Half-saturation of chemokines for T_reg_ recruitment | 0.5-5 (1.5) |
| *h_TregTNF_* | Half-saturation of TNF for T_reg_ recruitment | 0.05-0.5 (0.3) |
| *T_recTregMax_* | Maximum recruitment probability for T_reg_ recruitment | 0.008-0.06 (0.0221) |
| **Bacteria Related Parameters** | | |
| *α_Bi_* (per 10 min) | Intracellular Mtb growth rate | 5×10^-4^-5×10^-3^ (1.4×10^-3^) |
| *α_Be_* (per 10 min) | Extracellular Mtb growth rate | 10^-4^-10^-3^ (7×10^-4^) |
| *μ_BeCas_* | Maximum death rate of extracellular Mtb in caseation | 0.75-2 (1.5) |

^*^ Parameters used for sensitivity analysis are indicated by their ranges of values. Values in parentheses are used to generate the baseline containment parameter set [1,2].

**Table S4.** Molecular scale TNF/TNFR and IL10/IL10R parameters.

| **Parameter** | **Parameter group** | | **Parameter description** | **Value^*^** | **Reference** |
| --- | --- | --- | --- | --- | --- |
|  | | **TNF/TNFR Parameters** | | | |
| *k_synthMac_* (#/cell.s) | Synthesis | | Minimum synthesis rate of mTNF for macrophages | 10^-1^-1 (0.21) | [3,4] |
| *k_synthTcell_* (#/cell.s) | Synthesis | | Minimum synthesis rate of mTNF for T cells | 10^-2^-10^-1^ (0.021) | [3,4] |
| *k_trans_* (s^-1^) | Synthesis | | TNF mRNA translation rate constant | 10^-5^-10^-4^ (8.0x10^-5^) |  |
| *kRNA_Mac* (#/cell.s) | Synthesis | | Full TNF mRNA synthesis rate constant for macrophages | 1-5 (1.5) | [4–6] |
| *kRNA_Tcell* (#/cell.s) | Synthesis | | Full TNF mRNA synthesis rate constant for T cells | 0.1-0.5 (0.15) | [4–6] |
| *β_Mac_* | Synthesis | | Logistic function lower asymptote for macrophages that describes IL10-IL10R inhibition of TNF mRNA synthesis | *k_synthMac_ / kRNA_Mac* |  |
| *β_Tcell_* | Synthesis | | Logistic function lower asymptote for T cells that describes IL10-IL10R inhibition of TNF mRNA synthesis | *k_synthTcell_ / kRNA_Tcell* |  |
| *TNFR1_mac_* (#/cell) | Signaling | | TNFR1 density on the surface of macrophages | 500-5000 (1100-1900) ^†^ | [3,7–9] |
| *TNFR1_Tcell_* (#/cell) | Signaling | | TNFR1 density on the surface of T cells | 500-5000 (400-1200) ^†^ | [3,7–9] |
| *TNFR2_mac_* (#/cell) | Signaling | | TNFR2 density on the surface of macrophages | 500-5000 (400-800) ^†^ | [3,7–9] |
| *TNFR2_Tcell_* (#/cell) | Signaling | | TNFR2 density on the surface of T cells | 500-5000 (600-800) ^†^ | [3,7–9] |
| *D_TNF_* (cm^2^/s) ^‡^ | Spatial | | Diffusion coefficient of sTNF | 10^-8^-10^-7^ (5.2×10^-8^) | [10,11] |
| *D_sTNF/TNFR2_* (cm^2^/s) ^‡^ | Spatial | | Diffusion coefficient of shed TNF/TNFR2 complex | 10^-8^-10^-7^ (3.2×10^-8^) | [10,11] |
| *kTACE_Mac* (s^-1^) | Synthesis | | Rate constant for TNF release by TACE activity on a macrophage | 10^-4^-10^-3^ (4.4×10^-4^) | [3,12–14] |
| *kTACE_Tcell* (s^-1^) | Synthesis | | Rate constant for TNF release by TACE activity on a T cell | 10^-8^-10^-6^ (4.4×10^-5^) | [3,12–14] |
| *k_deg_* (s^-1^) | Spatial | | sTNF degradation rate constant | 5x10^-4^-5x10^-3^ (1.58×10^-4^) | [15] |
| *K_d1_* (M) | Signaling | | Equilibrium dissociation constant of sTNF/TNFR1 | 10^-12^-10^-10^ (1.9×10^-11^) | [7,16] |
| *K_d2_* (M) | Signaling | | Equilibrium dissociation constant of sTNF/TNFR2 | 10^-10^-10^-9^ (4.2×10^-10^) | [7,16,17] |
| *k_on1_* (M^-1^s^-1^) | Signaling | | sTNF/TNFR1 association rate constant | 10^7^-10^8^ (2.8×10^7^) | [16] |
| *k_on2_* (M^-1^s^-1^) | Signaling | | sTNF/TNFR2 association rate constant | 10^7^-10^8^ (3.5×10^7^) | [16] |
| *k_off1_* (s^-1^) | Signaling | | sTNF/TNFR1 dissociation rate constant | *k_on1_*×*K_d1_* |  |
| *k_off2_* (s^-1^) | Signaling | | sTNF/TNFR2 dissociation rate constant | *k_on2_*×*K_d2_* |  |
| *k_int1_* (s^-1^) | Signaling | | TNFR1 internalization rate constant | 1.5×10^-4^-1.5×10^-3^ (7.7×10^-4^) | [16,18] |
| *k_int2_* (s^-1^) | Signaling | | TNFR2 internalization rate constant | 3.9×10^-4^-5×10^-4^ (4.6×10^-4^) | [17] |
| *k_shed_* (s^-1^) | Spatial | | TNFR2 shedding rate constant | 3.9×10^-4^-1.5×10^-3^ (5×10^-4^) | [13,18] |
| *k_rec1_* (s^-1^) | Signaling | | TNFR1 recycling rate constant | 8.8×10^-5^-5.5×10^-4^ (1.8×10^-5^) | [19,20] |
| *k_rec2_* (s^-1^) | Signaling | | TNFR2 recycling rate constant | 8.8×10^-5^-5.5×10^-4^ (1.8×10^-5^) | [19,20] |
| *k_t1_* (s^-1^) | Signaling | | TNFR1 turn-over rate constant | 3×10^-4^-5×10^-4^ (3.8×10^-4^) | [19,20] |
| *k_t2_* (s^-1^) | Signaling | | TNFR2 turn-over rate constant | 3×10^-4^-5×10^-4^ (3.8×10^-4^) | [19,20] |
| *k_deg1_* (s^-1^) | Signaling | | TNFR1 degradation rate constant | 10^-5^-10^-4^ (5×10^-5^) | [7,19–21] |
| *k_deg2_* (s^-1^) | Signaling | | TNFR2 degradation rate constant | 10^-5^-10^-4^ (5×10^-5^) | [7,19–21] |
| *Vr1_mac* (#/cell.s) | Signaling | | Cell surface TNFR1 synthesis rate constant for macrophages | *k_t1_×TNFR1_mac_* |  |
| *Vr1_Tcell* (#/cell.s) | Signaling | | Cell surface TNFR1 synthesis rate constant for T cells | *k_t1_×TNFR1_Tcell_* |  |
| *Vr2_mac* (#/cell.s) | Signaling | | Cell surface TNFR2 synthesis rate constant for macrophages | *k_t2_×TNF21_mac_* |  |
| *Vr2_Tcell* (#/cell.s) | Signaling | | Cell surface TNFR2 synthesis rate constant for T cells | *k_t2_×TNF21_Tcell_* |  |
| *δ_I_* (# of IL10-IL10R/cell) | Signaling | | Inverse growth rate of logistic function that describes IL10-IL10R inhibition of TNF mRNA synthesis | 5-10 (7) | [6,22,23] |
| *γ_I_* | Signaling | | Time of max growth of logistic function that describes IL10-IL10R inhibition of TNF mRNA synthesis | 15-30 (20) | [6,22,23] |
|  | | **IL10/IL10R Parameters** | | | |
| *D_IL10_* (cm^2^/s) ^‡^ | Spatial | | Diffusion coefficient of soluble IL10 | 10^-8^-10^-7^ (5.2×10^-8^) | [10,11] |
| *K_d_* (M) | Signaling | | Equilibrium dissociation constant of IL10/IL10R | 10^-11^-10^-9^ (4.56x10^-10^) | [24–27] |
| *k_on_* (M^-1^s^-1^) | Signaling | | IL10/IL10R association rate constant | 10^5^-10^6^ (5.7x10^5^) | [24–27] |
| *k_off_* (s^-1^) | Signaling | | IL10/IL10R dissociation rate constant | *k_on_*×*K_d_* |  |
| *k_t_* (s^-1^) | Signaling | | IL10R turn-over rate constant | 5x10^-5^-5x10^-4^ (1.6x10^.4^) | [18,19,28] |
| *k_int_* (s^-1^) | Signaling | | IL10R internalization rate constant | 10^-4^-10^-3^ (5×10^-4^) | [29] |
| *k_deg_* (s^-1^) | Spatial | | Soluble IL10 degradation rate constant | 10^-4^-10^-3^ (4.8×10^-4^) | [30,31] |
| *Vr_mac* | Signaling | | Cell surface IL10R synthesis rate constant for macrophages | *k_t_×IL10R_mac_* |  |
| *Vr_Tcell* | Signaling | | Cell surface IL10R synthesis rate constant for Tcells | *k_t_×IL10R_Tcell_* |  |
| *IL10R_mac_* (#/cell) | Signaling | | IL10R density on the surface of macrophages | 500-5000 (1150-1850) ^†^ | [25–27,32,33] |
| *IL10R_Tcell_* (#/cell) | Signaling | | IL10R density on the surface of T cells | 100-1000 (250-550) ^†^ | [25–27,32,33] |
| *k_synthMacInf_* (#/cell.s) | Synthesis | | Full synthesis rate of soluble IL10 by infected macrophages | 0.01-0.1 (0.061) | [5,34] |
| *k_synthMacAct_* (#/cell.s) | Synthesis | | Full synthesis rate of soluble IL10 by activated macrophages | 0.1-1.0 (0.41) | [35,36] |
| *k_synthTcell_* (#/cell.s) | Synthesis | | Full synthesis rate of soluble IL10 by T cells | 0.25-1.5 (0.74) | [37–39] |
| *h_synthMacAct_* (# of sTNF-TNFR1/cell) | Synthesis | | Half-saturation of IL10 synthesis by activated macrophages induced by bound TNF to TNFR1 | 50-500 (190) | [35,36] |

^*^ Parameters used for sensitivity analysis are indicated by their ranges of values. Values in parentheses are used to generate the baseline containment parameter set.

^†^ Baseline containment parameter set values for TNFR and IL10R densities on each recruited individual cell were randomly chosen from the ranges shown in parentheses.

^‡^ Diffusion coefficients of soluble species in granuloma were estimated from values for diffusible factors of similar molecular weight in tumors [17,18].

**Table S5.** Molecular scale TNF-α induced cell response parameters.

| **Parameter** | **Parameter group** | **Parameter description** | **Value^*^** | **Reference** |
| --- | --- | --- | --- | --- |
| *k_NFκB_* ((#/cell)^-1^s^-1^) | Signaling | Rate constant for TNF-induced NFκB activation in macrophages | 8x10^-9^-3x10^-8^ (1.41×10^-8^) | [40] |
| *k_apopt_* ((#/cell)^-1^s^-1^) | Signaling | Rate constant for TNF-induced apoptosis in all cell types | 10^-10^-10^-9^ (3.45×10^-10^) |  |
| *τ_NFκB_* (#/cell) | Signaling | Cell surface sTNF/TNFR1 threshold for TNF-induced NFκB activation | 35-100 (65) | [40] |
| *τ_apopt_* (#/cell) | Signaling | Internalized sTNF/TNFR1 threshold for TNF-induced apoptosis | 1200-2300 (1728) |  |
| *s_apopt_* (#/cell) | Signaling | Saturation concentration of internalized sTNF/TNFR1 for TNF-induced apoptosis | 3500-4500 (4022) |  |
| *s_NFκB_* | Signaling | Saturation fraction of sTNF/TNFR1 for TNF-induced NFκB activation | 0.35-0.50 (0.43) | [40] |

^*^ Parameters used for sensitivity analysis are indicated by their ranges of values. Values in parentheses are used to generate the baseline containment parameter set.

**References**

1. Ray JCJ, Flynn JL, Kirschner DE (2009) Synergy between individual TNF-dependent functions determines granuloma performance for controlling Mycobacterium tuberculosis infection. Journal of immunology (Baltimore, Md : 1950) 182: 3706–3717. doi:10.4049/jimmunol.0802297.

2. Fallahi-Sichani M, El-Kebir M, Marino S, Kirschner DE, Linderman JJ (2011) Multiscale computational modeling reveals a critical role for TNF-α receptor 1 dynamics in tuberculosis granuloma formation. Journal of immunology (Baltimore, Md : 1950) 186: 3472–3483. doi:10.4049/jimmunol.1003299.

3. Fallahi-Sichani M, Schaller M a, Kirschner DE, Kunkel SL, Linderman JJ (2010) Identification of key processes that control tumor necrosis factor availability in a tuberculosis granuloma. PLoS computational biology 6: e1000778. doi:10.1371/journal.pcbi.1000778.

4. Marino S, Sud D, Plessner H, Lin PL, Chan J, et al. (2007) Differences in reactivation of tuberculosis induced from anti-TNF treatments are based on bioavailability in granulomatous tissue. PLoS computational biology 3: 1909–1924. doi:10.1371/journal.pcbi.0030194.

5. Giacomini E, Iona E, Ferroni L, Miettinen M, Fattorini L, et al. (2001) Infection of human macrophages and dendritic cells with Mycobacterium tuberculosis induces a differential cytokine gene expression that modulates T cell response. Journal of immunology (Baltimore, Md : 1950) 166: 7033–7041.

6. Smallie T, Ricchetti G, Horwood NJ (2010) IL-10 inhibits transcription elongation of the human TNF gene in primary macrophages. The Journal of Experimental Medicine. doi:10.1084/jem.20100414.

7. Imamura K, Spriggs D, Kufe D (1987) Expression of tumor necrosis factor receptors on human monocytes and internalization of receptor bound ligand. Journal of immunology (Baltimore, Md : 1950) 139: 2989–2992.

8. Pocsik E, Mihalik R, Ali-Osman F, Aggarwal BB (1994) Cell density-dependent regulation of cell surface expression of two types of human tumor necrosis factor receptors and its effect on cellular response. Journal of cellular biochemistry 54: 453–464. doi:10.1002/jcb.240540412.

9. Van Riemsdijk-Van Overbeeke IC, Baan CC, Knoop CJ, Loonen EH, Zietse R, et al. (2001) Quantitative flow cytometry shows activation of the TNF-alpha system but not of the IL-2 system at the single cell level in renal replacement therapy. Nephrology, dialysis, transplantation : official publication of the European Dialysis and Transplant Association - European Renal Association 16: 1430–1435.

10. Nugent LJ, Jain RK (1984) Extravascular diffusion in normal and neoplastic tissues. Cancer research 44: 238–244.

11. Pluen a, Boucher Y, Ramanujan S, McKee TD, Gohongi T, et al. (2001) Role of tumor-host interactions in interstitial diffusion of macromolecules: cranial vs. subcutaneous tumors. Proceedings of the National Academy of Sciences of the United States of America 98: 4628–4633. doi:10.1073/pnas.081626898.

12. Newton RC, Solomon K a, Covington MB, Decicco CP, Haley PJ, et al. (2001) Biology of TACE inhibition. Annals of the rheumatic diseases 60 Suppl 3: iii25–32.

13. Crowe PD, Walter BN, Mohler KM, Otten-Evans C, Black RA, et al. (1995) A metalloprotease inhibitor blocks shedding of the 80-kD TNF receptor and TNF processing in T lymphocytes. The Journal of experimental medicine 181: 1205–1210.

14. Solomon KA, Covington MB, DeCicco CP, Newton RC (1997) The fate of pro-TNF-alpha following inhibition of metalloprotease-dependent processing to soluble TNF-alpha in human monocytes. Journal of immunology (Baltimore, Md : 1950) 159: 4524–4531.

15. Cheong R, Bergmann A, Werner SL, Regal J, Hoffmann A, et al. (2006) Transient IkappaB kinase activity mediates temporal NF-kappaB dynamics in response to a wide range of tumor necrosis factor-alpha doses. The Journal of biological chemistry 281: 2945–2950. doi:10.1074/jbc.M510085200.

16. Grell M, Wajant H, Zimmermann G, Scheurich P (1998) The type 1 receptor (CD120a) is the high-affinity receptor for soluble tumor necrosis factor. Proceedings of the National Academy of Sciences of the United States of America 95: 570–575.

17. Pennica D, Lam VT, Mize NK, Weber RF, Lewis M, et al. (1992) Biochemical properties of the 75-kDa tumor necrosis factor receptor. Characterization of ligand binding, internalization, and receptor phosphorylation. The Journal of biological chemistry 267: 21172–21178.

18. Higuchi M, Aggarwal BB (1994) TNF induces internalization of the p60 receptor and shedding of the p80 receptor. Journal of immunology (Baltimore, Md : 1950) 152: 3550–3558.

19. Bajzer Z, Myers a C, Vuk-Pavlović S (1989) Binding, internalization, and intracellular processing of proteins interacting with recycling receptors. A kinetic analysis. The Journal of biological chemistry 264: 13623–13631.

20. Vuk-Pavlović S, Kovach JS (1989) Recycling of tumor necrosis factor-alpha receptor in MCF-7 cells. FASEB journal : official publication of the Federation of American Societies for Experimental Biology 3: 2633–2640.

21. Tsujimoto M, Yip YK, Vilcek J (1985) Tumor necrosis factor: specific binding and internalization in sensitive and resistant cells. Proceedings of the National Academy of Sciences of the United States of America 82: 7626–7630.

22. Brennan FM, Green P, Amjadi P, Robertshaw HJ, Alvarez-Iglesias M, et al. (2008) Interleukin-10 regulates TNF-α-converting enzyme (TACE/ADAM17) involving a TIMP3 dependent and independent mechanism. European Journal of Immunology 38: 1106–1117. doi:10.1002/eji.200737821.

23. Balcewicz-Sablinska M, Gan H, Remold HG (1999) Interleukin 10 produced by macrophages inoculated with Mycobacterium avium attenuates mycobacteria-induced apoptosis by reduction of TNF-α activity. The Journal of Infectious Diseases 180: 1230–1237.

24. Yoon S Il, Logsdon NJ, Sheikh F, Donnelly RP, Walter MR (2006) Conformational changes mediate interleukin-10 receptor 2 (IL-10R2) binding to IL-10 and assembly of the signaling complex. The Journal of biological chemistry 281: 35088–35096. doi:10.1074/jbc.M606791200.

25. Liu Y, Wei SH, Ho a S, De Waal Malefyt R, Moore KW (1994) Expression cloning and characterization of a human IL-10 receptor. Journal of immunology (Baltimore, Md : 1950) 152: 1821–1829.

26. Weber-Nordt RM, Meraz M a, Schreiber RD (1994) Lipopolysaccharide-dependent induction of IL-10 receptor expression on murine fibroblasts. Journal of immunology (Baltimore, Md : 1950) 153: 3734–3744.

27. Tan JC, Indelicato SR, Narula SK, Zavodny PJ, Chou CC (1993) Characterization of interleukin-10 receptors on human and mouse cells. The Journal of biological chemistry 268: 21053–21059.

28. Lauffenburger DA, Linderman JJ (1993) Receptors: Models For Binding, Trafficking, and Signaling New York: Oxford University Press.

29. Wei SH-Y, Ming-Lum A, Liu Y, Wallach D, Ong CJ, et al. (2006) Proteasome-mediated proteolysis of the interleukin-10 receptor is important for signal downregulation. Journal of interferon & cytokine research : the official journal of the International Society for Interferon and Cytokine Research 26: 281–290. doi:10.1089/jir.2006.26.281.

30. Moya C, Huang Z, Cheng P, Jayaraman A, Hahn J (2011) Investigation of IL-6 and IL-10 signalling via mathematical modelling. IET systems biology 5: 15. doi:10.1049/iet-syb.2009.0060.

31. Figueiredo AS, Höfer T, Klotz C, Sers C, Hartmann S, et al. (2009) Modelling and simulating interleukin-10 production and regulation by macrophages after stimulation with an immunomodulator of parasitic nematodes. The FEBS journal 276: 3454–3469. doi:10.1111/j.1742-4658.2009.07068.x.

32. Ho AS, Wei SH, Mui AL, Miyajima A, Moore KW, et al. (1995) Functional regions of the mouse interleukin-10 receptor cytoplasmic domain . These include : Functional Regions of the Mouse Interleukin-10 Receptor Cytoplasmic Domain. 15.

33. Ding Y, Qin L, Zamarin D, Sergei V, Pestka S, et al. (2012) Differential IL-10R1 Expression Plays a Critical Role in IL-10-Mediated Immune Regulation.

34. Shaw TC, Thomas LH, Friedland JS (2000) Regulation of IL-10 secretion after phagocytosis of Mycobacterium tuberculosis by human monocytic cells. Cytokine 12: 483–486. doi:10.1006/cyto.1999.0586.

35. Wu K, Koo J, Jiang X, Chen R, Cohen SN, et al. (2012) Improved control of tuberculosis and activation of macrophages in mice lacking protein kinase R. PloS one 7: e30512. doi:10.1371/journal.pone.0030512.

36. Verreck F a W, De Boer T, Langenberg DML, Hoeve M a, Kramer M, et al. (2004) Human IL-23-producing type 1 macrophages promote but IL-10-producing type 2 macrophages subvert immunity to (myco)bacteria. Proceedings of the National Academy of Sciences of the United States of America 101: 4560–4565. doi:10.1073/pnas.0400983101.

37. Yssel H, De Waal Malefyt R, Roncarolo MG, Abrams JS, Lahesmaa R, et al. (1992) IL-10 is produced by subsets of human CD4+ T cell clones and peripheral blood T cells. Journal of immunology (Baltimore, Md : 1950) 149: 2378–2384.

38. Meyaard L, Hovenkamp E, Otto SA, Miedema F (1996) IL-12-induced IL-10 production by human T cells as a negative feedback for IL-12-induced immune responses. Journal of immunology (Baltimore, Md : 1950) 156: 2776–2782.

39. Orme IM, Roberts AD, Griffin JP, Abrams JS (1993) Cytokine secretion by CD4 T lymphocytes acquired in response to Mycobacterium tuberculosis infection. Journal of immunology (Baltimore, Md : 1950) 151: 518–525.

40. Tay S, Hughey JJ, Lee TK, Lipniacki T, Quake SR, et al. (2010) Single-cell NF-kappaB dynamics reveal digital activation and analogue information processing. Nature 466: 267–271. doi:10.1038/nature09145.
