# Supplementary material for: Multi-Scale Modeling Predicts a Balance of Tumor Necrosis Factor-α and Interleukin-10 Controls the Granuloma Environment during Mycobacterium tuberculosis Infection
Source: PLoS One. 2013 Jul 15;8(7):e68680. doi: 10.1371/journal.pone.0068680 (PMC3711807; doi:10.1371/journal.pone.0068680)
Supplement: Appendix S4 — Supplemental Data and Figures. (DOCX) [file pone.0068680.s004.docx]

**Appendix S4 – Supplemental Data and Figures**

Herein, we present data for non-dominant outcomes briefly described in the main text. Our computational model is stochastic and therefore simulations are averaged over multiple replications in order to get a representation of model outputs. Although each simulation run is distinct there are several model outcomes that can occur: uncontrolled bacterial growth, bacterial containment, resolved bacterial clearance, and unresolved bacterial clearance. Resolved granulomas have no bacterial load and have returned to steady state values of Mϕ while unresolved granulomas have no bacterial load but contain Mϕ levels well above steady state values. These unresolved granulomas tend to have large amounts of tissue damage due to the persistence of activated Mϕ beyond clearance of bacterial load. In order to carry out common statistical techniques (such as mean and standard deviation) simulations must be grouped into outcomes to prevent single modal statistics from being calculated on a multi-modal distribution. Therefore, shown below are the non-dominant outcomes for simulation runs presented in the main text and figures.

**Table S6.** Signal Parameter Group – Receptor Expression – Non-Dominant Behavior Data.

|  |  | **Number of Simulations** | **Total Bacterial Load** | **Number of Activated Macrophages** | **Number of Apoptotic Resting Macrophages** |
| --- | --- | --- | --- | --- | --- |
| **Low** | *Macrophage TNFR Density* | 1 out of 30 | 0 ± N/A | 0 ± N/A | 0 ± N/A |
|  | *Macrophage IL-10R Density (Resolved Granulomas)^*^* | 5 out of 30 | 0 ± 0 | 0 ± 0 | 146 ± 178 |
|  | *Macrophage IL-10R Density (Unresolved Granulomas)^†^* | 5 out of 30 | 0 ± 0 | 25 ± 34 | 1557 ± 1157 |
| **Baseline** | *Baseline Parameters* | 2 out of 30 | 0 ± 0 | 0 ± 0 | 7 ± 1 |
| **High** | *Macrophage TNFR Density* | 6 out of 30 | 1141 ± 397 | 100 ± 44 | 301 ± 66 |
|  | *Macrophage IL-10R Density* | 0 out of 30 | - | - | - |

* Resolved Granulomas – Levels of resting and activated Mϕ have returned to steady state values

† Unresolved Granulomas - Levels of resting and activated Mϕ have not returned to steady state values

**Table S7.** Spatial Parameter Group – Non-Dominant Behavior Data.

|  |  | **Number of Simulations** | **Total Bacterial Load** | **Number of Activated Macrophages** | **Number of Apoptotic Resting Macrophages** |
| --- | --- | --- | --- | --- | --- |
| **Low** | *TNF-α Spatial Influence* | 1 out of 30 | 0 ± N/A | 0 ± N/A | 0 ± N/A |
|  | *IL-10 Spatial Influence (Resolved Granulomas) ^*^* | 6 out of 30 | 0 ± 0 | 0 ± 0 | 746 ± 225 |
|  | *IL-10 Spatial Influence (Unresolved Granulomas) ^†^* | 8 out of 30 | 0 ± 0 | 479 ± 247 | 3325 ± 1267 |
| **Baseline** | *Baseline Parameters* | 2 out of 30 | 0 ± 0 | 0 ± 0 | 7 ± 1 |
| **High** | *TNF-α Spatial Influence* | 0 out of 30 | - | - | - |
|  | *IL-10 Spatial Influence* | 1 out of 30 | 0 ± N/A | 0 ± N/A | 0 ± N/A |

* Resolved Granulomas – Levels of resting and activated Mϕ have returned to steady state values

† Unresolved Granulomas - Levels of resting and activated Mϕ have not returned to steady state values

**Table S8.** Signal Parameter Group – Internalization Rate – Non-Dominant Behavior Data.

|  |  | **Number of Simulations** | **Total Bacterial Load** | **Number of Activated Macrophages** | **Number of Apoptotic Resting Macrophages** |
| --- | --- | --- | --- | --- | --- |
| **Low** | *TNF-α Internalization Rate* | 7 out of 30 | 165 ± 174 | 2906 ± 145 | 0 ± 0 |
|  | *IL-10 Internalization Rate* | 3 out of 30 | 0 ± 0 | 0 ± 0 | 3 ± 2 |
| **Baseline** | *Baseline Parameters* | 2 out of 30 | 0 ± 0 | 0 ± 0 | 7 ± 1 |
| **High** | *TNF-α Internalization Rate* | 3 out of 30 | 0 ± 0 | 0 ± 0 | 13 ± 7 |
|  | *IL-10 Internalization Rate* | 9 out of 30 | 0 ± 0 | 0 ± 0 | 106 ± 116 |

**Table S9.** Synthesis Parameter Group – Non-Dominant Behavior Data.

|  |  | **Number of Simulations** | **Total Bacterial Load** | **Number of Activated Macrophages** | **Number of Apoptotic Resting Macrophages** |
| --- | --- | --- | --- | --- | --- |
| **Low** | *TNF-α Synthesis* | 0 out of 30 | - | - | - |
|  | *IL-10 Synthesis (Resolved) ^*^* | 4 out of 30 | 0 ± 0 | 0 ± 0 | 121 ± 200 |
|  | *IL-10 Synthesis (Unresolved) ^†^* | 7 out of 30 | 0 ± 0 | 146± 213 | 1719 ± 1083 |
| **Baseline** | *Baseline Parameters* | 2 out of 30 | 0 ± 0 | 0 ± 0 | 7 ± 1 |
| **High** | *TNF-α Synthesis* | 13 out of 30 | 362 ± 302 | 1040 ± 135 | 4630 ± 576 |
|  | *IL-10 Synthesis* | 1 out of 30 | 0 ± N/A | 0 ± N/A | 0 ± N/A |

* Resolved Granulomas – Levels of resting and activated Mϕ have returned to steady state values

† Unresolved Granulomas - Levels of resting and activated Mϕ have not returned to steady state values

**Table S10.** IL-10 Knockout Parameter Set – Non-Dominant Behavior Data.

|  |  | **Number of Simulations** | **Total Bacterial Load** | **Number of Activated Macrophages** | **Number of Apoptotic Resting Macrophages** |
| --- | --- | --- | --- | --- | --- |
| **Day 200** | *Resolved* | 7 out of 50 | 0 ± 0 | 0 ± 0 | 382 ± 189 |
|  | *Unresolved* | 17 out of 50 | 3 ± 7 | 146 ± 177 | 1931 ± 724 |

* Resolved Granulomas – Levels of resting and activated Mϕ have returned to steady state values

† Unresolved Granulomas - Levels of resting and activated Mϕ have not returned to steady state values

**Table S11.** Uncertainty and Sensitivity Analysis Results For the Effects of TNF and IL-10 Molecular Scale Parameters on Model Outputs at 200 Days Post-Infection.

|  | **TNF-Related Parameters** | | | | | | | | | | | | | | **IL10 Related Parameters** | | | | | | |
| --- | --- | --- | --- | --- | --- | --- | --- | --- | --- | --- | --- | --- | --- | --- | --- | --- | --- | --- | --- | --- | --- |
|  | D_TNF_ | τ_apop_ | k_SynthMac_ | k_RNA_Mac_ | K_d1_ | k_on1_ | k_int1_ | k_rec1_ | k_T2_ | k_deg_ | k_deg1_ | TNFR1_Mac_ | τ_NFkB_ | k_NFkB_ | K_d_ | k_on_ | k_int_ | k_deg_ | IL10R_Mac_ | D_IL10_ | k_SynthMacAct_ |
| **TNF-Induced Outputs** |  |  |  |  |  |  |  |  |  |  |  |  |  |  |  |  |  |  |  |  |  |
| Apoptosis – Mac |  | --- |  | +++ | --- | +++ | + | --- |  | --- | -- | +++ |  |  | + | --- | +++ |  | --- | ++ | --- |
| Apoptosis – Mr |  | -- |  | +++ | --- |  |  | --- |  | --- |  |  |  |  |  | -- | +++ |  | --- |  | --- |
| Apoptosis – Mi |  | -- |  | +++ | --- | + | +++ | --- |  | -- | - | ++ |  |  |  | - | + |  |  |  |  |
| Apoptosis – Mci |  | --- |  | +++ |  |  | +++ | --- | - | - |  | + |  |  |  |  |  |  |  |  |  |
| Apoptosis – Ma |  | --- |  | +++ | --- | ++ |  | --- |  | --- | -- | +++ |  |  | + | --- | +++ |  | --- | +++ | --- |
| Apoptosis – T cells |  | -- |  | +++ | -- |  |  | --- |  | - | - | --- |  |  |  | - | +++ |  | --- |  | -- |
| NFkB – Mr | +++ |  | + | +++ | -- |  | --- |  |  | --- |  |  | --- | +++ |  | --- | +++ | ++ | --- |  | --- |
| NFkB – Mi |  |  |  |  |  |  |  |  |  |  |  |  |  |  |  |  |  |  | ++ |  | ++ |
| **Cellular-Level Outputs** |  |  |  |  |  |  |  |  |  |  |  |  |  |  |  |  |  |  |  |  |  |
| IntMtb |  | +++ | - | --- | +++ | --- | +++ |  | - | + |  | --- |  | --- | --- | +++ | --- | - | +++ |  | +++ |
| ExtMtb |  | ++ | -- | --- | +++ | --- | +++ |  | - | ++ |  | --- |  | --- | -- | +++ | --- |  | +++ |  | +++ |
| TotMtb |  | ++ | -- | --- | +++ | --- | +++ |  | - | ++ |  | --- |  | --- | -- | +++ | --- |  | +++ |  | +++ |
| Mr | + |  |  |  |  |  | --- |  |  |  |  | - | -- |  |  |  | + |  |  |  | - |
| Mi |  | +++ | - | --- | +++ | --- | +++ |  | -- | + |  | --- |  | --- | -- | +++ | --- |  | +++ |  | +++ |
| Mci |  | +++ | - | --- | +++ | --- | +++ |  | - | + |  | --- |  | --- | --- | +++ | --- | - | +++ |  | +++ |
| Ma | +++ |  |  |  |  |  | --- |  |  | -- |  |  | --- | + |  | --- | ++ |  | --- |  | -- |
| Tgam | +++ |  |  |  |  |  | --- |  |  | - |  |  | -- |  |  |  |  |  |  |  |  |
| Tcyt | +++ |  |  |  |  |  | --- |  |  | - |  |  | -- |  |  |  |  |  |  |  | - |
| Treg | +++ |  |  |  |  |  | --- |  |  | --- |  |  | - |  |  |  | + |  | - |  | - |
| **Tissue-Level Outputs** |  |  |  |  |  |  |  |  |  |  |  |  |  |  |  |  |  |  |  |  |  |
| Caseation |  |  |  |  |  | - | +++ |  |  |  |  |  |  |  |  |  |  |  | +++ |  |  |
| Granuloma Size | +++ |  |  |  |  |  | --- |  |  |  |  | - | - |  |  |  |  |  |  |  | - |
| [TNF] | +++ |  | + | +++ |  |  | --- |  |  | --- |  | --- | -- |  |  | --- | +++ | ++ | --- |  | --- |
| [IL10] | ++ |  |  |  |  |  | --- |  |  | - |  |  | -- |  |  | --- | + | --- | --- |  |  |
| [Chemokines] | +++ |  |  |  |  |  | --- |  |  | - |  |  | --- |  |  | - |  |  | --- |  |  |

Significant PRCC values are as follows: -/+ 0.001 < p < 0.01 --/++ 0.0001 < p < 0.001 ---/+++ p < 0.0001

**Table S12.** Uncertainty and Sensitivity Analysis Results For the Effects of Cellular Scale Parameters on Model Outputs at 200 Days Post-Infection.

|  | **Chemokine Parameters** | | **T Cell Parameters** | | | | **Recruitment Parameters** | | | | | | | | **Bacterial Parameters** | |
| --- | --- | --- | --- | --- | --- | --- | --- | --- | --- | --- | --- | --- | --- | --- | --- | --- |
|  | D_Chem_ | k_degChem_ | P_STAT1_ | P_Fas/FasL_ | P_CytKill_ | T_MoveM_ | M_recMex_ | h_MacTNF_ | h_MacCC_ | T_recTgamMax_ | τ_recTgamTNF_ | T_recTcytMax_ | T_recTregMax_ | τ_recTregTNF_ | α_Bi_ | α_Be_ |
| **TNF-Induced Outputs** |  |  |  |  |  |  |  |  |  |  |  |  |  |  |  |  |
| Apoptosis – Mac | +++ | --- | ++ | --- | - | -- | + | --- | --- |  |  |  | -- |  |  |  |
| Apoptosis – Mr | +++ | --- | ++ | --- |  | -- | + | --- | --- |  |  |  | --- |  |  |  |
| Apoptosis – Mi | +++ | --- |  | --- | -- | -- | ++ | --- | --- |  |  |  |  |  |  |  |
| Apoptosis – Mci | +++ | --- |  | --- | --- | --- | + | --- | --- |  |  | -- |  |  | ++ |  |
| Apoptosis – Ma | +++ | --- | +++ | --- |  | - |  | - | -- | + |  |  | --- | + | + |  |
| Apoptosis – T cells | +++ | --- |  | --- | --- |  |  | --- | --- |  |  |  | --- |  | +++ |  |
| NFkB – Mr | +++ | --- | ++ | --- |  | -- | + | --- | --- |  |  |  | --- | + |  |  |
| NFkB – Mi | +++ | --- |  | --- | -- | -- | ++ | --- | --- |  |  |  | - |  | + |  |
| **Cellular-Level Outputs** |  |  |  |  |  |  |  |  |  |  |  |  |  |  |  |  |
| IntMtb | +++ | --- |  | --- | -- | -- | + | -- | --- |  | + | -- |  | + | + |  |
| ExtMtb | +++ | --- | -- | --- | --- | --- | ++ | -- | --- | -- | + | --- | ++ |  | +++ | ++ |
| TotMtb | +++ | --- | -- | --- | --- | --- | ++ | -- | --- | -- | + | --- | ++ |  | +++ | + |
| Mr | +++ | --- |  |  |  |  |  | - | - |  |  |  |  |  | --- |  |
| Mi | +++ | --- |  | --- | - | - | + | --- | -- |  |  | -- |  |  | +++ |  |
| Mci | +++ | --- |  | --- | - | -- |  | -- | --- |  | + | -- |  | + |  |  |
| Ma | +++ | --- | +++ | --- |  | - |  | - | -- |  |  |  | --- | + |  |  |
| Tgam | +++ | --- |  | --- |  | - |  |  |  | +++ |  |  |  |  | +++ |  |
| Tcyt | +++ | --- |  | --- | --- | - |  | - |  |  |  | +++ |  |  | +++ |  |
| Treg | +++ | --- |  | --- | - | - |  | - |  | - |  | -- | +++ |  | +++ |  |
| **Tissue-Level Outputs** |  |  |  |  |  |  |  |  |  |  |  |  |  |  |  |  |
| Caseation |  | --- |  |  |  |  |  | -- |  |  |  |  |  |  | +++ | + |
| Granuloma Size | +++ | --- |  | --- | --- | --- | +++ | --- | --- |  | + | --- |  |  | +++ |  |
| [TNF] | +++ | --- | + | --- | - | -- | + | -- | --- |  |  | - |  | + | + |  |
| [IL10] | +++ | --- | + | --- |  | - |  | - | - |  |  |  |  |  | +++ |  |
| [Chemokines] | +++ | --- | + | --- |  | -- |  | - | - |  |  |  |  |  | + |  |

Significant PRCC values are as follows: -/+ 0.001 < p < 0.01 --/++ 0.0001 < p < 0.001 ---/+++ p < 0.0001


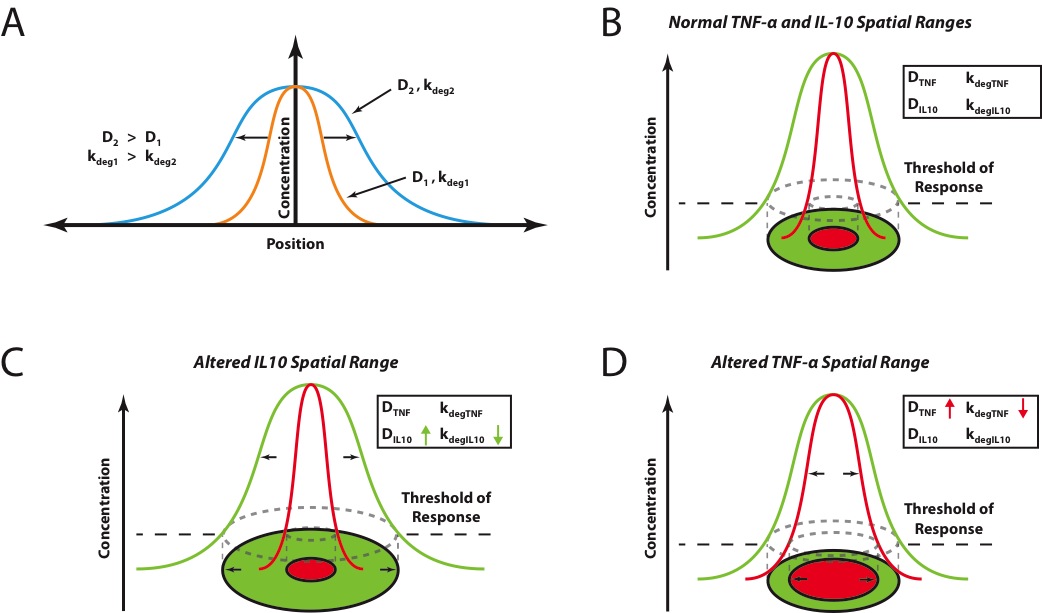


**Figure S3.** **Schematic diagram showing how the spatial ranges of TNF-α and IL-10 are manipulated.** A. As the diffusivity is increased and the degradation rate is decreased the spatial range of the molecule is increased (blue curve vs. the orange curve). B. Representation of the spatial range of TNF-α (red curve) and IL-10 (green curve) using the baseline parameters (Table S2 in Appendix S3). C. Increasing the diffusivity and decreasing the degradation rate constants of IL-10 increases the spatial range of IL-10 (green curve). D. Increasing the diffusivity and decreasing the degradation rate constants of TNF-α increases the spatial range of TNF-α (red curve).

**Figure S4.**  **Solution of the coupled IL-10 and TNF-α ODEs.** IL-10 and TNF-α ODE solutions for 1x10^6^ cells/mL at various initial exogenous IL-10 conditions.
